# Elevations in Donor-Derived Cell-Free DNA and Allograft Outcomes in Kidney Transplantation

**DOI:** 10.1681/ASN.0000001133

**Published:** 2026-06-16

**Authors:** Jeffrey A. Klein, David Taber, David Wojciechowski, Didier A. Mandelbrot, Renata Glehn-Ponsirenas, Oyedolamu Olaitan, Pranjal Jain, James Lu, Martina M. McGrath, Mohanram Narayanan, Anup M. Patel, Massini Merzkani, Nitipong Permpalung, Rashikh A. Choudhury, Michelle A. Josephson, Sanjiv Anand, Pradeep Vaitla, Eric Langewisch, Maria Ajaimy, Dhiren Kumar, George W. Burke, Todd E. Pesavento, Anthony J. Langone, Yihung Huang, David Leeser, Bernard Fischbach, Nadiesda Costa, Kalathil Sureshkumar, Abd A. Qannus, Chun-Po Steve Fan, Ziad Zaky, Fuad Shihab, Sanjay Kulkarni, Thin Thin Maw, Bekir Tanriover, Enver Akalin, Matthew Cooper, Ibrahim Tawhari, Matthew R. Weir, Tarek Alhamad, Daniel C. Brennan, Nicolae Leca, Suphamai Bunnapradist, Ling Shen, Robert N. Woodward, Peale Chuang, Prince Anand, Emilio D. Poggio, Giselle Guerra, Alexander Wiseman, Anil Paramesh, Diane Cibrik, Jonathan Bromberg

**Affiliations:** 1 Division of Nephrology and Hypertension, Department of Internal Medicine, University of Kansas, Kansas City, Kansas; 2 Department of Surgery, Medical University of South Carolina, Charleston, South Carolina; 3 Department of Internal Medicine, University of Texas Southwestern, Dallas, Texas; 4 Department of Medicine, University of Wisconsin Hospital, Madison, Wisconsin; 5 CareDx, Inc, Brisbane, California; 6 Department of Surgery, Rush University Medical Center, Chicago, Illinois; 7 Tampa General Hospital, University of South Florida, Tampa, Florida; 8 Brigham and Women's Hospital, Boston, Massachusetts; 9 Division of Nephrology and Hypertension, Department of Medicine, Baylor Scott and White Medical Center, Temple, Texas; 10 Cooperman Barnabas Medical Center, Livingston, New Jersey; 11 Division of Nephrology, Washington University in St. Louis, St. Louis, Missouri; 12 Department of Medicine, Johns Hopkins University School of Medicine, Baltimore, Maryland; 13 Department of Surgery–Transplant, University of Colorado Hospital, Denver, Colorado; 14 Department of Medicine, University of Chicago, Chicago, Illinois; 15 Intermountain Healthcare, Salt Lake City, Utah; 16 Division of Nephrology, University of Mississippi Medical Center, Jackson, Mississippi; 17 Department of Internal Medicine, Division of Nephrology, University of Nebraska, Omaha, Nebraska; 18 Montefiore-Einstein Center for Transplantation, Montefiore Medical Center, Bronx, New York; 19 Division of Nephrology, Department of Internal Medicine, Virginia Commonwealth University, Richmond, Virginia; 20 University of Miami Miller School of Medicine, Miami Transplant Institute, Miami, Florida; 21 Department of Internal Medicine, Ohio State University, Columbus, Ohio; 22 Vanderbilt University Medical Center, Nashville, Tennessee; 23 Division of Transplant Nephrology, UC Davis Health System, Davis, California; 24 ECU/Vidant Medical Center, Greenville, North Carolina; 25 Baylor University Medical Center, Dallas, Texas; 26 MedStar Georgetown Transplant Institute, Washington, DC; 27 Division of Nephrology and Hypertension, Medicine Institute, Allegheny General Hospital, Pittsburgh, Pennsylvania; 28 Division of Nephrology, Department of Medicine, The University of Arizona, Tucson, Arizona; 29 Department of Kidney Medicine, Cleveland Clinic, Cleveland, Ohio; 30 Division of Nephrology, University of Utah, Salt Lake City, Utah; 31 Yale University School of Medicine, New Haven, Connecticut; 32 Division of Nephrology and Hypertension, Keck Medicine of USC, Los Angeles, California; 33 Medical College of Wisconsin, Milwaukee, Wisconsin; 34 Division of Nephrology, Department of Medicine, College of Medicine, University of Illinois, Chicago, Illinois; 35 Division of Nephrology, Department of Medicine, University of Maryland School of Medicine, Baltimore, Maryland; 36 Division of Nephrology, University of Washignton, Seattle, Washington; 37 Division of Nephrology, David Geffen School of Medicine, Los Angeles, California; 38 Medical University of South Carolina, Lancaster, South Carolina; 39 AdventHealth Transplant Institute, Denver, Colorado; 40 Tulane University School of Medicine, New Orleans, Louisiana

**Keywords:** immunology, kidney biopsy, kidney dysfunction, kidney failure, kidney transplantation, mortality, outcomes, rejection, survival

## Abstract

**Key Points:**

- We assessed whether an initial donor-derived cell-free DNA elevation during routine surveillance was associated with allograft dysfunction and loss in kidney transplant recipients.
- Intermediate and high donor-derived cell-free DNA states were independently associated with 3.7-fold and 6.4-fold higher risks of allograft loss, respectively.
- Persistently low donor-derived cell-free DNA levels were associated with low rates of rejection and allograft loss, identifying a low-risk population.

**Background:**

Although donor-derived cell-free DNA (dd-cfDNA) correlates with histologic rejection and injury severity, its association with long-term graft outcomes has yet to be well established in kidney transplant recipients. We hypothesize that among patients monitored with dd-cfDNA, an initial elevation may be associated with subsequent allograft dysfunction and loss.

**Methods:**

The Kidney Allograft Outcomes AlloSure Registry is a prospective, multicenter cohort of 1743 adult kidney transplant recipients from 56 US centers; this analysis included 1258 patients who initiated dd-cfDNA surveillance 15–60 days post-transplant with up to 3-year follow-up. A multistate model was applied to characterize transitions from a low dd-cfDNA state (no elevations) to intermediate (0.5%–1.0% with ≥61% relative change value) or high (≥1.0%) dd-cfDNA states, and their unadjusted and covariate-adjusted associations with allograft loss or all-cause death were quantified using hazard regression models.

**Results:**

Within 3 years, 36% of patients transitioned to an elevated dd-cfDNA state (intermediate *n*=216; high *n*=233). Allograft dysfunction by 36 months occurred significantly more often in patients with dd-cfDNA elevations despite preserved allograft function at the time of elevation (eGFR ≥60 ml/min per 1.73 m^2^ in 42% of intermediate and 33% of high), consistent with subclinical injury. Transitions from low to elevated dd-cfDNA states were strongly associated with subsequent allograft loss (adjusted hazard ratios, 3.70 [95% confidence interval [CI], 1.38 to 9.90] for intermediate; 6.36 [95% CI, 2.80 to 14.42] for high), while mortality did not differ by dd-cfDNA state.

**Conclusions:**

Initial dd-cfDNA elevations exceeding established thresholds were independently associated with higher risks of allograft dysfunction and loss across both elevation categories, whereas patients who remained in the low dd-cfDNA state experienced favorable outcomes.

## Visual Abstract

**Figure:**
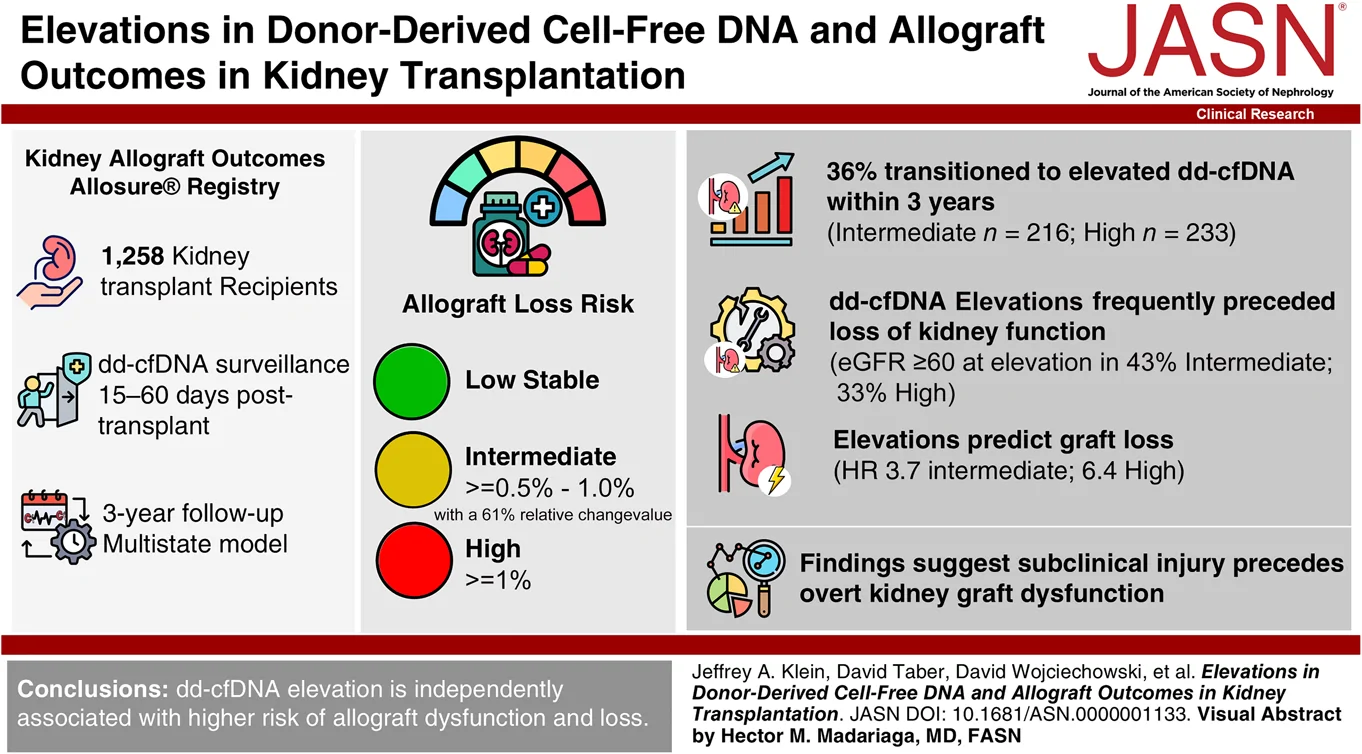

## Introduction

Kidney transplantation remains the preferred treatment for kidney failure, offering superior survival and quality of life compared with dialysis. Recent analyses of the US national transplant registry data show incremental gains in long-term allograft survival among both deceased and living donor kidney transplants.^1,2^ However, chronic immune-mediated injury remains the leading cause of allograft failure. This chronic injury is primarily driven by ongoing antibody-meditated and *T*-cell–mediated rejection (TCMR) leading to progressive microvascular injury and fibrosis, highlighting the need for better tools and therapies to address these immune processes.^1,2^

Despite strong interest in identifying new noninvasive biomarkers to improve the management of patients who received a kidney transplant, none have demonstrated the ability to predict rejection and other important graft outcomes when compared with other standard-of-care parameters. A recent systematic review found that most candidate biomarkers lack sufficient clinical validation.^3^ Studies of blood transcriptomics and non-HLA antibody profiles, like the European Transplantation and Innovation trial, failed to demonstrate additive value beyond conventional markers for rejection detection or outcome prediction.^4^ Recently, microvascular inflammation has been recognized as a key mechanism of chronic allograft injury, even in donor-specific antibodies (DSA)–negative recipients, underscoring the need for more sensitive and precise surveillance tools that can identify microvascular inflammation in the absence of DSA.^5,6^

Among the available biomarkers, only donor-derived cell-free DNA (dd-cfDNA) has demonstrated a strong correlation with the severity of histologic injury and with acute rejection, including subclinical rejection.^7–10^ Because this assay has been available for clinical use, dd-cfDNA has facilitated transplant rejection surveillance, monitoring treatment responses, differentiating the clinical significance of ambiguous rejection phenotypes, and avoiding unnecessary biopsies.^10–13^ A recent large multinational study, which included a well-phenotyped cohort of nearly 4000 kidney transplant recipients, confirmed that dd-cfDNA levels independently correlate with the degree of allograft inflammation and injury.^8^ Whether dd-cfDNA levels are associated with long-term allograft survival has not yet been established.

The Kidney Allograft Outcomes AlloSure Registry (KOAR; NCT03326076) is a prospective, observational, multicenter registry designed to evaluate clinical outcomes in adult kidney transplant recipients whose post-transplant management incorporates dd-cfDNA surveillance.

The objective was to evaluate the association between a patient's initial dd-cfDNA elevation and subsequent clinical outcomes, regardless of when this occurred during the follow-up period and without consideration of later dd-cfDNA levels.

## Methods

### Study Design and Setting

KOAR is a prospective, observational, multicenter cohort study conducted at 56 kidney transplant centers in the United States. Adult kidney transplant recipients were enrolled between January 2018 and December 2020 and followed for 3 years post-transplant.

The study protocol recommended dd-cfDNA surveillance at 1, 2, 3, 4, and 6 months post-transplantation and quarterly thereafter, with additional testing performed as clinically indicated. Testing frequency was determined by treating clinicians according to the institutional protocols and medical necessity.

The study protocol was approved by the Western Institutional Review Board and local institutional review boards at the participating sites. Participants provided written informed consent before enrollment. Study coordination, data management, and statistical analyses were performed by the study sponsor.

### Participants

This analysis included patients who received a kidney transplant (*de novo* or repeat) and had a dd-cfDNA test performed between 15 and 60 days post-transplant. Patients were excluded if they experienced rejection or allograft loss before their first dd-cfDNA, were pregnant, received a multiorgan transplant or a kidney from a monozygotic twin, or had a history of hematopoietic stem cell transplantation.

### Data Sources and Measurements

Clinical data included donor and recipient demographics, transplant characteristics, immunosuppressive regimens, and longitudinal follow-up assessments.

#### dd-cfDNA Quatification

Blood samples were collected in Streck Cell-Free DNA blood collection tubes and analyzed using the *AlloSure* next-generation sequencing assay, as described previously.^7,14^

#### dd-cfDNA States

All patients were assigned to the low state after transplantation. dd-cfDNA results were evaluated to identify the first test at which a result met criteria for transition to the intermediate or high state. Once a patient transitioned to the intermediate or high state, they remained in that state for all subsequent analyses, reflecting real-world clinical management as future dd-cfDNA levels cannot be predicted at the time of a given test. Patients for whom the first dd-cfDNA result was ≥1.0% transitioned directly from the low to high state. The states were defined as follows.

- Low dd-cfDNA state (baseline): Initial state after transplantation for all patients; patients without dd-cfDNA elevation remained in the low state.
- Intermediate dd-cfDNA state: dd-cfDNA elevation between ≥0.5% and <1.0% with a ≥61% relative change value from the prior test.
- High dd-cfDNA state: dd-cfDNA elevation ≥1.0%.

#### Biopsy Data

Local pathology reports were collected for all kidney allograft biopsies and centrally reviewed for Banff criteria alignment by a nephropathologist blinded to the dd-cfDNA results. Diagnoses were standardized according to the Banff 2019 criteria^15^ and categorized as no rejection, TCMR, antibody-mediated rejection (ABMR), and mixed rejection.

#### Clinical Outcomes

The primary outcome was allograft loss at 3 years post-transplant, defined as permanent return to dialysis or retransplantation. Secondary outcomes were all-cause mortality and evidence of allograft dysfunction, defined as eGFR ≤30 ml/min per 1.73 m^2^ (CKD Epidemiology Collaboration equation) at 12, 24, and 36 months post-transplant.

### Statistical Analysis

#### Descriptive Statistics

For the descriptive analysis, patients who transitioned to intermediate or high dd-cfDNA states were categorized as intermediate or high groups, whereas patients who remained in the low dd-cfDNA state were considered the low group. Grouping definition is described in the time-to-event section. Baseline characteristics were summarized using standard descriptive statistics. Categorical variables are presented as counts and percentages, and continuous variables are presented as means ± standard deviation (SD) or medians with interquartile range (IQR), as appropriate.

The cumulative incidence of allograft loss was estimated using the Gray subdistribution method, and the mortality was characterized using the Kaplan–Meier survival method. The respective 95% confidence intervals (CIs) for the 3-year cumulative incidence rates of allograft loss and mortality were calculated on the log scale and back-transformed to the absolute risk scale.

The number of dd-cfDNA tests per patient during the first post-transplant year was assessed and characterized using Andersen–Gill models. To evaluate the association between dd-cfDNA elevation and biopsy results, analyses were restricted to the first biopsy obtained after dd-cfDNA elevation, or for patients without dd-cfDNA elevation, the first biopsy following initiation of dd-cfDNA surveillance.

#### Time-to-Event Analysis

Evaluating post-transplant risk requires analytical methods that account for complex, time-varying processes in which multiple events can contribute to allograft failure or death.^16^ A multistate model was applied to assess the association between a patient's initial dd-cfDNA elevation with subsequent clinical outcomes.

The multistate model represents each patient's post-transplant course according to their first qualifying dd-cfDNA elevation state, with death modeled as a competing risk. ^17^ Figure 1A illustrates the analytic schema, depicting transitions among dd-cfDNA states with allograft loss, with death treated as a competing terminal end point.

**Figure 1:**
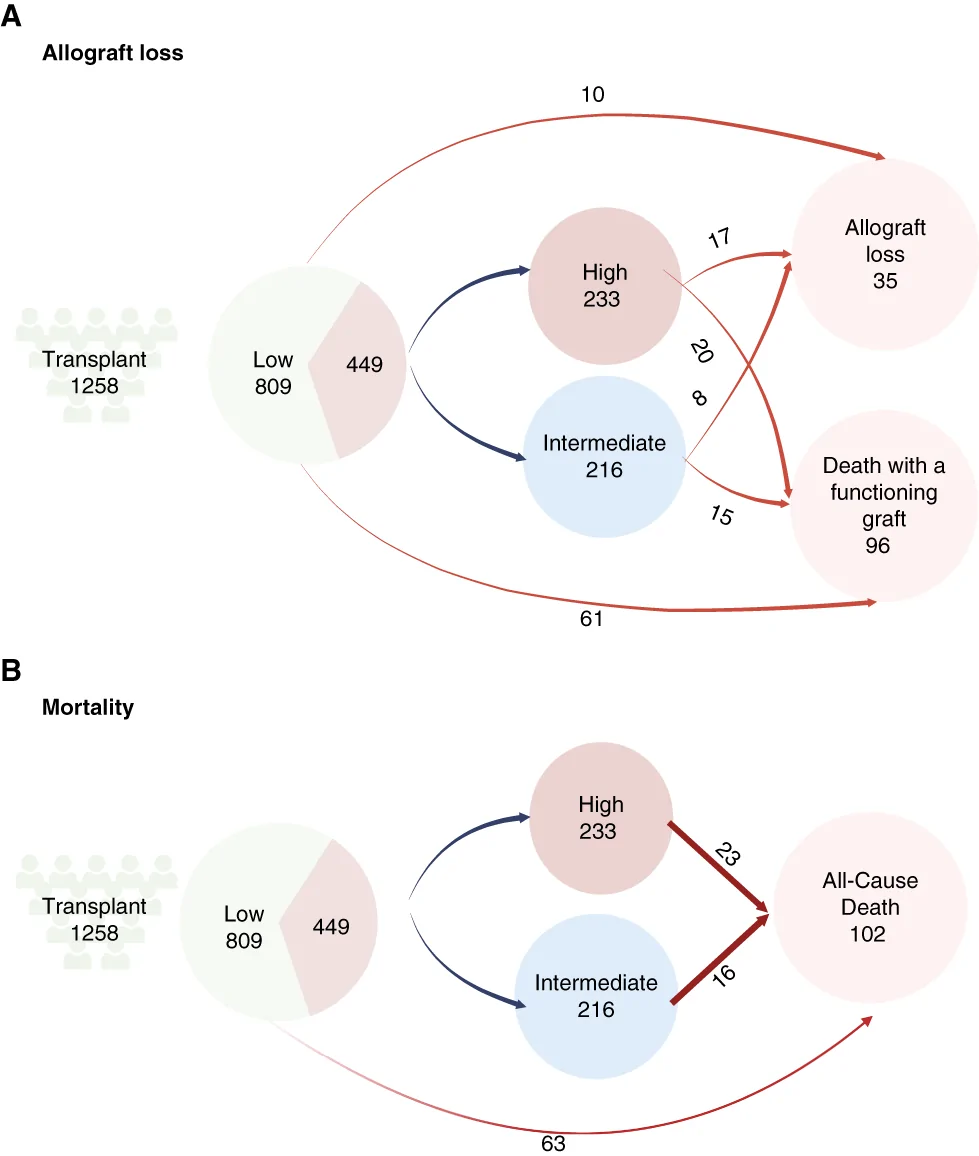
**Multistate model scheme of dd-cfDNA elevation states.** All patients entered the model in the low dd-cfDNA state after transplantation. On first qualifying elevation, patients transitioned once to either the intermediate (≥0.5% and <1% with ≥61% relative change value; *n*=216) or high (≥1%; *n*=233) state and remained there for the remainder of follow-up; patients without elevation (*n*=809) remained in the low state. Transitions were unidirectional. (A) Allograft loss. After elevation, patients could move only to a terminal state—allograft loss (*n*=35) or death with a functioning graft (*n*=96)—whichever occurred first; only the first terminal event was counted. Death without allograft loss was treated as a competing risk. (B) Mortality. All-cause mortality was *n*=102 (low *n*=63, intermediate *n*=16, high *n*=23), regardless of allograft status. dd-cfDNA, donor-derived cell-free DNA.

The multistate model assumes that all patients started their post-transplant journey in the low dd-cfDNA state until their first elevated dd-cfDNA measurement, allograft loss, or death, whichever happened first. Each patient could move to either the intermediate or high state, depending on the extent of the elevation as defined above. Transitioning to graft loss or death could occur from low/intermediate/high dd-cfDNA states. Note that the multistate model assumes the transitions be unidirectional (*e.g*., no return to low dd-cfDNA state once in high dd-cfDNA state) as we consider initial elevation in dd-cfDNA informative of the allograft loss risk. Supplemental Figure 1 illustrates the patient journey through a series of examples.

We also illustrate the relation between dd-cfDNA states and all-cause mortality using the multistate model schema (Figure 1B).

We conducted unadjusted and covariate-adjusted regression to quantify the association between dd-cfDNA status and a clinical outcome. In the unadjusted analyses for allograft loss, extended cause-specific hazard regression was applied to estimate the cause-specific hazard ratios (HRs) of the (time-varying) dd-cfDNA elevation. The rationale to use this regression is to assess the association of dd-cfDNA elevation specifically with allograft loss, whereas death was considered as a competing risk. In the unadjusted analysis for all-cause mortality, extended Cox hazard regression was applied to estimate HRs of dd-cfDNA elevation. In the covariate-adjusted analyses for both outcomes, we started with the unadjusted model and proceeded with the covariate selection using a forward stepwise approach with a model entry criterion of χ^2^ *P* values < 0.05. The candidate covariates under consideration included donor and recipient characteristics, such as demographic, immunologic, and transplant-related factors.

Finally, to assess the robustness of the primary findings, we performed sensitivity analyses, treating dd-cfDNA value as a time-varying continuous covariate, using the aforementioned extended hazard regression. In addition, landmark analyses were performed at 3, 6, 12, and 18 months post-transplant, by classifying patients by histories of dd-cfDNA elevation status before each landmark and quantifying the association of the dd-cfDNA status with subsequent allograft loss.

All analyses were performed using SAS version 9.4 (SAS Institute, Cary, NC) and *R* version 4.4.2 with *R* packages MASS 7.2, mstate 0.3.3, cmprsk 2.2, survival 3.7.

## Results

### Study Population and Baseline Characteristics

Of the 1743 kidney transplant recipients enrolled in KOAR, 1258 met the inclusion criteria for analysis (Figure 2). The baseline characteristics of the KOAR cohort were generally representative of the national kidney transplant population reported in the Scientific Registry of Transplant Recipients for the same transplant period (Supplemental Table 1). The median follow-up duration was 1097 (1038–1101) days.

**Figure 2:**
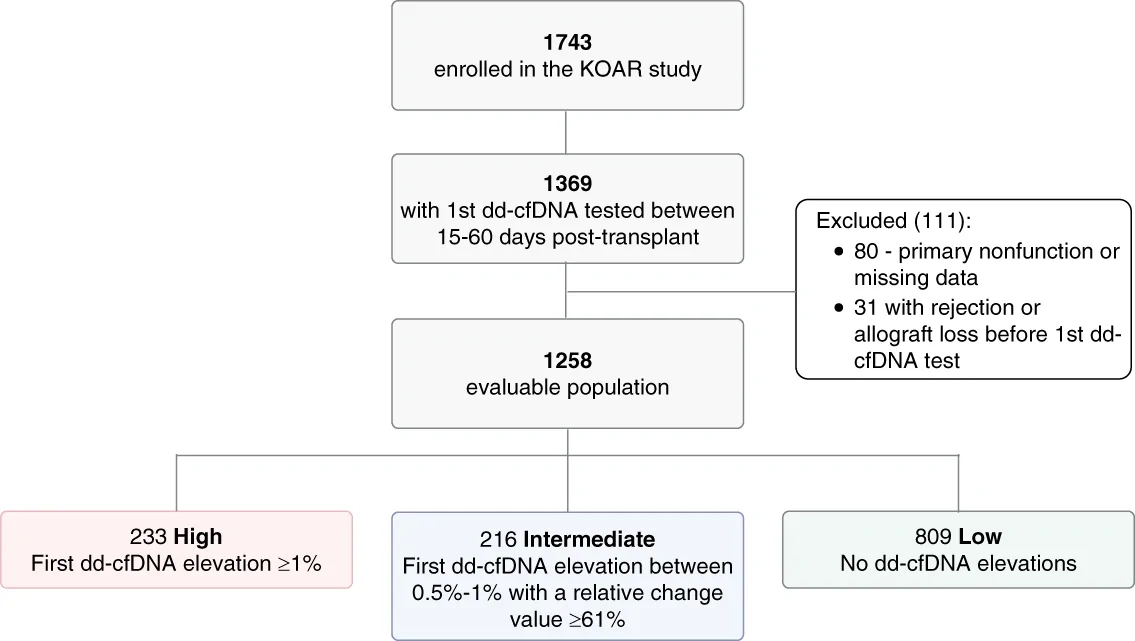
**Study population flow chart.** KOAR enrolled 1743 kidney transplant recipients. This analysis was restricted to 1258 patients who had a first dd-cfDNA test between 15 and 60 days post-transplant and at risk for rejection and allograft loss at the dd-cfDNA surveillance initiation. KOAR, Kidney Allograft Outcomes AlloSure Registry.

During follow-up, 449 (36%) patients experienced dd-cfDNA elevation and transitioned to either the intermediate (*n*=216; 17%) or high state (*n*=233; 19%), whereas 809 (64%) patients remained in the low state. For descriptive summaries, patients were categorized into low, intermediate, or high dd-cfDNA groups based on their first dd-cfDNA elevation, if one occurred.

Baseline characteristics were similar across the dd-cfDNA groups (Table 1), although patients in the intermediate and high groups were more likely to be sensitized or to have received a prior transplant than those who remained in the low group. Sensitization calculated panel reactive antibodies (cPRA) ≥80% was observed in 24% (*n*=51) of the intermediate and 27% (*n*=63) of the high groups, versus 9% (*n*=76) in the low group. Similarly, 19% (*n*=41) of the intermediate and 22% (*n*=51) of the high groups were repeat transplant recipients, compared with 6% (*n*=47) of the low group.

**Table 1:** Baseline characteristics by donor-derived cell-free DNA elevation group

Characteristic	Total (*N*=1258)	Low (*n*=809)	Intermediate (*n*=216)	High (*n*=233)
**Recipient characteristics**				
Age, mean±SD	55±13	55±13	52±14	53±13
Age category, No*.* (%)				
*19–29*	66 (5)	35 (4)	18 (8)	13 (6)
*30–39*	158 (13)	87 (11)	35 (16)	36 (15)
*40–49*	201 (16)	128 (16)	29 (13)	44 (19)
*50–59*	303 (24)	193 (24)	58 (27)	52 (22)
*60–69*	418 (33)	291 (36)	58 (27)	69 (30)
*≥70*	112 (9)	75 (9)	18 (8)	19 (8)
Sex, No*.* (%)				
*Male*	780 (62)	521 (64)	121 (56)	138 (59)
*Female*	478 (38)	288 (36)	95 (44)	95 (41)
Race, No*.* (%)				
*Asian*	78 (6)	57 (7)	9 (4)	12 (5)
*Black*	392 (31)	248 (31)	68 (31)	76 (33)
*Other*	102 (8)	61 (8)	24 (11)	17 (7)
*Unknown*	31 (2)	15 (2)	10 (5)	6 (3)
*White*	655 (52)	428 (53)	105 (49)	122 (52)
Cause of kidney transplant, No*.* (%)				
*Diabetic kidney disease*	386 (31)	269 (33)	56 (26)	61 (26)
*FSGS*	33 (3)	19 (2)	5 (2)	9 (4)
*Genetic*	108 (9)	68 (8)	20 (9)	20 (9)
*GN*	250 (20)	145 (18)	51 (24)	54 (23)
*HIV nephropathy*	7 (1)	4 (0)	1 (0)	2 (1)
*Hypertension*	305 (24)	203 (25)	48 (22)	54 (23)
*Unclear/other*	169 (13)	101 (13)	35 (16)	33 (14)
Dialysis at the time of transplant, No*.* (%)				
*No*	108 (9)	69 (9)	20 (9)	19 (8)
*Yes*	1148 (91)	738 (91)	196 (91)	214 (92)
Prior transplant, No*.* (%)				
*No*	1119 (89)	762 (94)	175 (81)	182 (78)
*Yes*	139 (11)	47 (6)	41 (19)	51 (22)
HLA mismatches, median [IQR]	4 [3–5]	4 [3–5]	5 [3–5]	4 [3–5]
cPRA (%), No*.* (%)				
*<80%*	953 (76)	657 (81)	145 (67)	151 (65)
*≥80%*	190 (15)	76 (9)	51 (24)	63 (27)
Pretransplant DSA, No*.* (%)^a^				
*Positive*	84 (7)	47 (6)	16 (7)	21 (9)
*Negative/unknown*	1174 (93)	762 (94)	200 (93)	212 (91)
Induction therapy, No*.* (%)				
*ATG-based*	760 (60)	480 (59)	132 (61)	148 (64)
*Basiliximab-based*	167 (13)	122 (15)	25 (12)	20 (9)
*Alemtuzumab-based*	213 (17)	124 (15)	46 (21)	43 (18)
*Other/mixed*	110 (9)	77 (10)	11 (5)	22 (9)
*Unknown/not documented*	8 (1)	6 (1)	2 (1)	0 (0)
Maintenance immunosuppression at mo 4, No*.* (%)				
*Tacrolimus-based*	1170 (93)	765 (95)	196 (91)	209 (90)
*Belatacept-based*	18 (1)	7 (1)	6 (3)	5 (2)
*Other/mixed*	38 (3)	15 (2)	11 (5)	12 (5)
*Missing*	32 (3)	22 (3)	3 (1)	7 (3)
**Donor characteristics**				
Donor age, mean±SD	41.2±15.4	42.3±15.2	40.4±14.8	38.2±16.3
Donor sex, No*.* (%)				
*Male*	605 (48)	378 (47)	105 (49)	122 (52)
*Female*	530 (42)	342 (42)	94 (44)	94 (40)
*Not provided*	123 (10)	89 (11)	17 (8)	17 (7)
Cold ischemia time (hours), mean±SD	14.2±9.8	14.0±9.7	13.8±10.3	15.6±9.4
KDPI, No*.* (%)				
*Living donor*	275 (22)	180 (22)	56 (26)	39 (17)
*Deceased donor*, *KDPI <20%*	147 (12)	89 (11)	21 (10)	37 (16)
*Deceased donor*, *KDPI 21%–85%*	686 (55)	436 (54)	119 (55)	131 (56)
*Deceased donor*, *KDPI >85%*	116 (9)	82 (10)	13 (6)	21 (9)
*Deceased donor*, *KDPI missing*	34 (3)	22 (3)	7 (3)	5 (2)
DGF, No*.* (%)^b^				
*No*	1076 (86)	687 (85)	192 (89)	197 (85)
*Yes*	182 (14)	122 (15)	24 (11)	36 (15)

ATG, antithymocyte globulin; cPRA, calculated panel reactive antibodies; DGF, delayed graft function; DSA, donor-specific antibody; HLA, human leukocyte antigen; IQR, interquartile range; KDPI, Kidney Donor Profile Index.

a Pretransplant donor-specific antibody information was based on the presence of a positive virtual or physical crossmatch.

b Delayed graft function rates include all donors (living and deceased).

### dd-cfDNA Testing Patterns and Elevation Dynamics

During the first-year post-transplant, participants had an average of 5.6 (95% CI, 5.5 to 5.7) dd-cfDNA tests. Adjusted mean testing rates were higher among patients who later transitioned to the elevated groups: 5.3 (95% CI, 5.2 to 5.6; low), 6.3 (95% CI, 5.9 to 6.6; intermediate), and 6.1 (95% CI, 5.7 to 6.4; high) tests per patient (*P* < 0.001). The median time to the first dd-cfDNA elevation was 340 (IQR, 182–584) days in the intermediate group and 189 (IQR, 97–317) days in the high group. Most dd-cfDNA elevations occurred within the first post-transplant year: 65% (intermediate) and 85% (high) (Figure 3).

**Figure 3:**
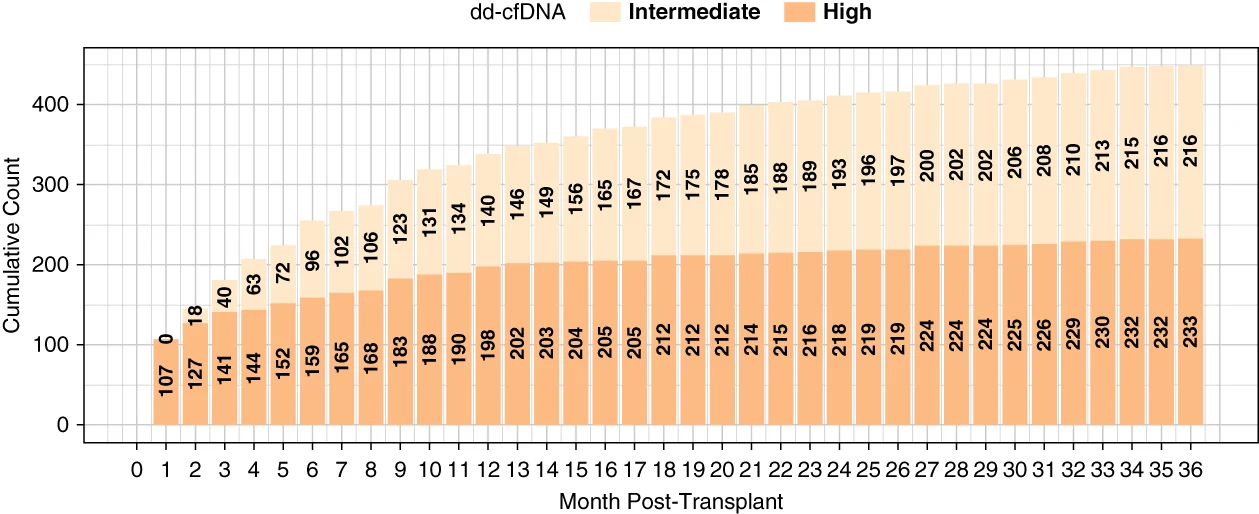
**Cumulative incidence of dd-cfDNA elevations during 3-year post-transplant follow-up.** Shows the cumulative count of first dd-cfDNA elevation during the 36-month follow-up. Elevations are categorized as high (dd-cfDNA ≥1.0%) or intermediate (dd-cfDNA between 0.5% and 1.0% with ≥61% relative change increase from prior value). Each vertical bar represents the total number of patients reaching an elevation by that month, with numerical counts displayed for both categories.

### Biopsy Utilization and Histologic Findings

The biopsy frequency varied across dd-cfDNA groups. The proportion of patients undergoing at least one biopsy was 37% in the low (296/809), 30% in the intermediate (65/216), and 60% in the high (139/233) groups (*P* < 0.001) (Supplemental Table 2). Among biopsied patients, biopsy-proven rejection (TCMR and ABMR) occurred in 4% of the low (32/809), 17% of the intermediate (37/216), and 27% of the high (62/233) (*P* < 0.001) groups. The median time from dd-cfDNA elevation to biopsy was 97 (IQR, 37–265) days in the intermediate group and 25 (IQR, 13–102) days in the high group.

Rejection phenotypes also differed across dd-cfDNA groups. In the first biopsy obtained after dd-cfDNA elevation (or the first biopsy ever for the low group), TCMR accounted for 94% (16/17) of rejections in the low and 75% (15/20) in the intermediate groups. In the high group, 48% (24/50) of rejections were categorized as ABMR or mixed. Among biopsies classified as no rejection by Banff 2019 criteria, nonrejection histopathologic findings were identified across all three dd-cfDNA groups and are detailed in Supplemental Table 3.

### Allograft Function

Allograft dysfunction, defined as eGFR ≤30 ml/min per 1.73 m^2^, was comparable across groups early after transplantation. At month 1, allograft dysfunction occurred in 14%, 15%, and 16% of patients in the low, intermediate, and high groups, respectively (*P* = 0.77); at month 3, the rates were 6%, 7%, and 6%, respectively (*P* = 0.86).

After 12 months, allograft dysfunction diverged across the dd-cfDNA groups (Figure 4) and increased progressively over time in all three groups. At month 12, allograft dysfunction occurred in 4%, 10%, and 10% of patients in the low, intermediate, and high groups, respectively, increasing to 8%, 11%, and 19% at month 36 among patients with available eGFR data (*P* < 0.001). At the time of dd-cfDNA elevation, 43% of intermediate and 33% of high group patients maintained eGFR ≥60 ml/min per 1.73 m^2^, indicating that most elevations occurred before significant decline of kidney function (Supplemental Figure 2). These findings were consistent when restricted to the 599 patients (low *N*=375, intermediate *N*=115, and high *N*=109) with complete eGFR data at both month 12 and month 36; Supplemental Figure 3.

**Figure 4:**
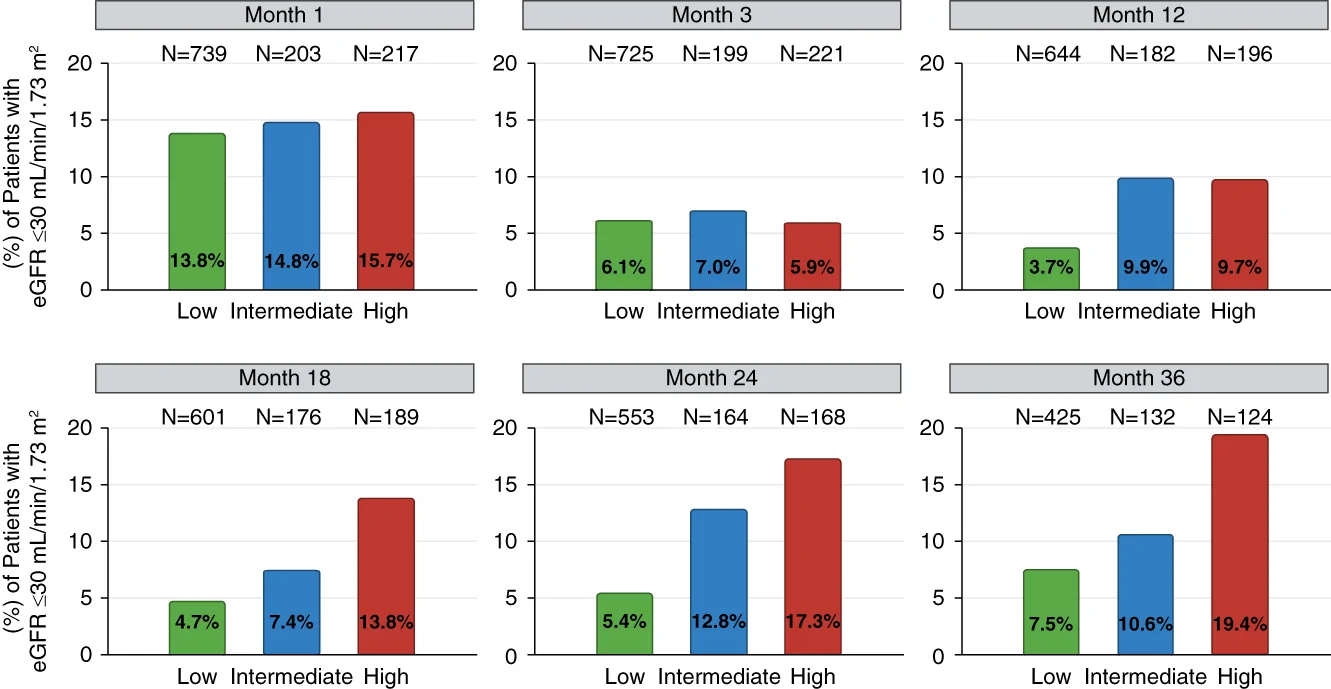
**Proportion of patients with allograft dysfunction by dd-cfDNA group.** Proportion of patients with eGFR ≤30 ml/min per 1.73 m^2^ at months 1, 3, 12, 18, 24, and 36, by dd-cfDNA group (low, intermediate, and high). Intermediate and high dd-cfDNA groups consisted of patients who transitioned to intermediate and high dd-cfDNA states upon their first dd-cfDNA elevation.

### Allograft Loss

Thirty-five (3%) patients experienced allograft loss during the 3-year follow-up. The 3-year cumulative incidence of allograft loss was significantly higher across the dd-cfDNA groups: 1.4% (95% CI, 0.7% to 2.6%) in the low, 3.9% (95% CI, 2.0% to 7.7%) in the intermediate, and 7.6% (95% CI, 4.8% to 12.1%) in the high groups (Table 2).

**Table 2:** Three-year cumulative incidence of allograft loss by donor-derived cell-free DNA group

dd-cfDNA Group	Allograft Loss (No.)	Incidence Rate (%; 95% CI)
Low	10	1.4% (0.7% to 2.6%)
Intermediate	8	3.9% (2.0% to 7.7%)
High	17	7.6% (4.8% to 12.1%)

The 95% confidence interval constructed on the log scale and back-transformed to the absolute risk scale. CI, confidence interval; dd-cfDNA, donor-derived cell-free DNA.

Compared with the low state, the unadjusted HR for allograft loss was 4.56 (95% CI, 1.71 to 12.16) for intermediate and 8.10 (95% CI, 3.60 to 18.22) for high states. After adjusting for pretransplant factors (cPRA ≥80% and retransplantation), these associations remained significant: HR, 3.70 (95% CI, 1.38 to 9.90) for intermediate and HR, 6.36 (95% CI, 2.80 to 14.42) for high (Table 3). Among the eight patients in the intermediate state who experienced allograft loss, four (50%) had dd-cfDNA ≥1% after their initial elevation before losing their grafts.

**Table 3:** Cause-specific hazard regression for 3-year allograft loss

Variable	Unadjusted Cause-Specific HR (95% CI)	Covariate-Adjusted Cause-Specific HR (95% CI)
**dd-cfDNA status** ^a^		
Intermediate versus low	4.56 (1.71 to 12.16)	3.70 (1.38 to 9.90)
High versus low	8.10 (3.60 to 18.22)	6.36 (2.80 to 14.42)
**cPRA**		
≥80% versus <80%		1.53 (1.22 to 1.93)
**Transplant**		
Repeated versus *de novo* transplant		1.93 (1.52 to 2.45)

CI, confidence interval; cPRA, calculated panel reactive antibodies; dd-cfDNA, donor-derived cell-free DNA; HR, hazard ratio.

a Death was modeled as a competing risk.

When modeling dd-cfDNA as a time-varying continuous covariate, higher values remained significantly associated with allograft loss. Landmark analyses substantiate the association of dd-cfDNA states with allograft loss across all chosen time points, primarily among patients transitioning to the high state. These sensitivity analyses support the robustness of the primary findings (Supplemental Tables 4 and 5). Causes of allograft loss by dd-cfDNA state are summarized in Supplemental Table 6.

### Mortality

During the 3-year follow-up, 102 (8%) patients died; six deaths occurred after allograft loss, and approximately one third were coronavirus disease 2019–related. The 3-year cumulative mortality did not differ significantly among the dd-cfDNA groups (Table 4): 8.6% (95% CI, 6.8% to 10.9%) in the low, 7.9% (95% CI, 4.9% to 12.6%) in the intermediate, and 10.9% (95% CI, 7.4% to 16.0%) in the high groups.

**Table 4:** Three-year cumulative incidence of mortality by donor-derived cell-free DNA group

dd-cfDNA Group	Number of Deaths (No.)	Incidence Rate (%; 95% CI)
Low	63	8.6% (6.8% to 10.9%)
Intermediate	16	7.9% (4.9% to 12.6%)
High	23	10.9% (7.4% to 16.0%)

The 95% confidence interval constructed on the log scale and back-transformed to the absolute risk scale. CI, confidence interval; dd-cfDNA, donor-derived cell-free DNA.

Transitions to intermediate or high dd-cfDNA states were not significantly associated with mortality in extended Cox regression analyses. The unadjusted HRs were 1.34 (95% CI, 0.77 to 2.33) for the intermediate and 1.61 (95% CI, 1.00 to 2.61) for high. After adjustment for cPRA ≥80% and repeat transplant, the adjusted HRs were 1.08 (95% CI, 0.60 to 1.92) for the intermediate group and 1.26 (95% CI, 0.76 to 2.08) for the high group (Table 5).

**Table 5:** Hazard regression for 3-year mortality

Variable	Unadjusted HR (95% CI)	Covariate-Adjusted HR (95% CI)^a^
**dd-cfDNA status**		
Intermediate versus low	1.34 (0.77 to 2.33)	1.08 (0.60 to 1.92)
High versus low	1.61 (1.00 to 2.61)	1.26 (0.76 to 2.08)

CI, confidence interval; dd-cfDNA, donor derived cell-free DNA; HR, hazard ratio.

a Model adjusted for calculated panel reactive antibodies ≥80% and repeat transplant.

## Discussion

In this large, prospective, multicenter cohort of kidney transplant recipients, we demonstrate that patients experiencing a dd-cfDNA elevation at any point during the 3-year follow-up were at a higher risk of subsequent allograft dysfunction and loss, with risk increasing with the magnitude of that elevation. The higher risk of allograft failure among those transitioning from low to either intermediate or high dd-cfDNA states was independent of baseline demographic and immunologic risk factors. These findings suggest that a single dd-cfDNA elevation represents a clinically meaningful shift in patient risk status, underscoring the prognostic value of routine dd-cfDNA surveillance. Importantly, the cohort of patients whose dd-cfDNA levels remained consistently low had favorable outcomes with less kidney dysfunction and low rates of allograft loss.

Our analyses included patients with elevated dd-cfDNA levels throughout the 3-year follow-up period, with a median time to first elevation of 340 days in the intermediate group and 189 days in the high group. These findings support the benefit of dd-cfDNA surveillance beyond the first post-transplant year, a time when prognostic noninvasive testing is particularly useful as most kidney transplant recipients transition from specialized transplant centers to community nephrology care.^18^ In such settings, dd-cfDNA offers a standardized and objective biomarker for monitoring allograft health, even across diverse clinical environments. Notably, in the intermediate group, a subset of patients later exceeded the 1% dd-cfDNA threshold, after which they had a higher risk of allograft loss, consistent with the prognostic significance of serial dd-cfDNA monitoring beyond a single measurement. These findings align with previous studies demonstrating that dd-cfDNA reflects the severity of Banff histologic lesions and serves as a marker of allograft injury, and biopsy guided by dd-cfDNA improves diagnostic yield by enriching for histologic rejection while reducing unnecessary procedures in low-risk patients.^7,8,10^

Biopsies in this study were classified according to Banff 2019 criteria,^15^ and cases with microvascular inflammation DSA-negative C4d-negative, probable ABMR, or borderline TCMR were included in the no rejection category, reflecting the prevailing interpretive practices across most transplant centers in current care. This may explain why a subset of patients with elevated dd-cfDNA levels had biopsies that were classified as negative for rejection. This distinction is clinically relevant, as Sablik *et al*. recently demonstrated that microvascular inflammation without DSA and C4d is associated with inferior allograft survival and subsequent development of ABMR.^5^ Moreover, published data suggest that dd-cfDNA may predict allograft injury earlier than histologic changes or functional decline and provide a complementary surveillance tool by identifying patients with subclinical injury who may warrant closer follow-up or intervention.^8–10,19^

A minority of dd-cfDNA elevations may reflect conditions other than rejection, although this likely differs according to the kidney transplant population of interest. For example, in pediatric cohorts, dd-cfDNA elevations have been reported to be associated with polyomavirus nephropathy;^20^ however, the results were not consistent in a multicenter study that included both adult and pediatric cohorts,^8^ indicating that the overlap between viral infection and alloimmune injury may complicate interpretation.^20–23^ Molecular transcriptomic studies have similarly identified rejection-associated gene expression patterns in biopsies without diagnostic histological changes, underscoring that conventional morphology may underestimate immune activity.^19,24,25^

Of the 1258 kidney transplant recipients, 809 (64%) remained in the low dd-cfDNA group. These patients were found to have only a 4% rejection and 1.2% graft loss rate over the 3-year follow-up period, which exemplifies the strong negative predictive value of dd-cfDNA. Despite having lower rejection and allograft loss events, cumulative mortality rates were similar across groups, suggesting that factors outside the alloimmune pathway, including comorbidities, contributed to patient mortality.^26–28^ These findings also highlight the opportunity to refine immunosuppressive regimens in clinically stable transplant recipients to reduce the burden of immunosuppression. Strategies such as conversion from calcineurin inhibitors to belatacept have demonstrated long-term functional benefits but carry a higher risk of acute rejection,^29,30^ whereas reduction of antiproliferative agents may mitigate the risk of infection, malignancy, and other adverse effects associated with cumulative immunosuppressive exposure.^31–33^ In this context, dd-cfDNA may serve a dual role by identifying patients suitable for immunosuppression minimization and enabling early detection of subclinical alloimmune injury during immunosuppression reduction. Recent literature also emphasizes the need for sensitive, noninvasive tools to guide precise immunosuppression and differentiate ambiguous clinical phenotypes, including cases of microvascular inflammation in the absence of DSA.^5,34,35^

The strength of this study is that it is one of the largest multicenter prospective studies conducted on kidney transplantation with a study cohort that is representative of the contemporary US kidney transplant population. However, there are several limitations worthy of discussion. First, as an observational registry without a mandated dd-cfDNA testing or biopsy schedule, clinical practices, including surveillance frequency, biopsy decisions, and response to dd-cfDNA elevations, varied across centers and likely evolved over time, particularly during the early dd-cfDNA adoption phase. Clinical decisions made after dd-cfDNA elevation, such as biopsy decisions or changes in immunosuppression, were not completely captured in the dataset, limiting insights into how dd-cfDNA elevations may have influenced outcomes. The analysis therefore could not distinguish outcomes among patients with successfully treated rejection, refractory rejection, or no identified rejection, which would have likely influenced the outcomes in those with elevated dd-cfDNA. Even so, transitioning to an elevated dd-cfDNA state was consistently associated with worse clinical outcomes, indicating that risk stratification based on an initial dd-cfDNA elevation remained informative in this cohort. Second, completeness of eGFR data decreased progressively over follow-up (81% at month 12; 54% at month 36); therefore, findings at later time points reflect only patients with available data. Third, the overall event rate for allograft loss was low but consistent with event rates in modern transplant practice,^1^ which limited the power to conduct detailed subgroup analyses. Finally, the study period overlapped with the coronavirus disease 2019 pandemic, which likely influenced clinical management including follow-up intensity and biopsy practices.

In conclusion, dd-cfDNA elevation was independently associated with a clinically meaningful higher risk of allograft loss. Elevated risk was observed across elevation categories, with serial monitoring providing prognostic value beyond isolated measurements. Conversely, patients who remained in the low dd-cfDNA state experienced favorable outcomes throughout follow-up, suggesting a complementary role for dd-cfDNA in identifying both higher-risk patients who may warrant closer evaluation and lower-risk patients who may be candidates for immunosuppression optimization.

## Supplementary Material

## Data Availability

Data belong to a third party, and authors are not authorized to share the data. Identity of Third Party: Study sponsor: CareDx. Reason for Restriction: The data underlying this study are derived from the Kidney Allograft Outcomes AlloSure Registry (KOAR; NCT03326076), a prospective, sponsor-managed, multicenter observational registry conducted under institutional review board–approved protocols and subject to patient confidentiality and data use restrictions. Because the dataset contains protected health information and is governed by site-specific and sponsor data-sharing agreements, it cannot be made publicly available. Qualified researchers may submit a reasonable request for data access to the study sponsor (CareDx, Inc.) for evaluation in accordance with applicable privacy, ethical, and regulatory requirements.

## Declarative Statements

This is an observatuonal study and received Institutional Review Board or Ethics Committee approval. All patients provided written informed consent. This is an obervational study and complies with the Declaration of Helsinki. The clinical and research activities being reported are consistent with the Principles of the Declaration of Istanbul as outlined in the Declaration of Istanbul on Organ Trafficking and Transplant Tourism.

## Supplemental Material

Supplemental Figure 1. Representative patient trajectories according to dd-cfDNA state.

Supplemental Figure 2. Longitudinal eGFR trajectories by dd-cfDNA group and CKD stage over 36 months.

Supplemental Figure 3. Allograft dysfunction by dd-cfDNA group in patients with complete eGFR data through year 3.

Supplemental Table 1. Comparison of baseline characteristics between KOAR study cohort and National SRTR Kidney Transplant Recipients (2018–2020).

Supplemental Table 2. Biopsy results according to first biopsy.

Supplemental Table 3. Histopathologic findings among biopsies classified as no rejection, by dd-cfDNA group.

Supplemental Table 4. Cause-specific hazard regression with dd-cfDNA as a time-varying, continuous covariate.

Supplemental Table 5. Landmark analysis to estimate the HR of 3-year allograft loss at 3, 6, 12, and 18 months post-transplant.

Supplemental Table 6. Causes of allograft loss by dd-cfDNA group.
