# Supplementary material for: Elevations in Donor-Derived Cell-Free DNA and Allograft Outcomes in Kidney Transplantation
Source: J Am Soc Nephrol. 2026 Jun 16;37(11):2517–28. doi: 10.1681/ASN.0000001133 (PMC13742986; doi:10.1681/ASN.0000001133)
Supplement: Supplementary file 1 [file jasn-37-2517-s001.pdf]

ASN American Society of Nephrology
JASN Journal of the American Society of Nephrology
KIDNEY360 Accessing Our World From Every Angle
CJASN Clinical Journal of the American Society of Nephrology

ASN Journal Disclosure Form

As per ASN journal policy, I have disclosed any financial relationships or commitments I have held in the past 36 months as included below. I have listed my Current Employer below to indicate there is a relationship requiring disclosure. If no relationship exists, my Current Employer is not listed.

M. Ajaimy reports the following:
Employer: Montefiore Medical Center

I understand that the information above will be published within the journal article, if accepted, and that failure to comply and/or to accurately and completely report the potential financial conflicts of interest could lead to the following: 1) Prior to publication, article rejection, or 2) Post-publication, sanctions ranging from, but not limited to, issuing a correction, reporting the inaccurate information to the authors' institution, banning authors from submitting work to ASN journals for varying lengths of time, and/or retraction of the published work.

Name: Maria Ajaimy
Manuscript ID: JASN-2025-001600R1
Manuscript Title: Elevations in Donor-Derived Cell-Free DNA Predict Allograft Outcomes in Kidney Transplantation
Date of Completion: April 23, 2026
Disclosure Updated Date: April 23, 2026

ASN American Society of Nephrology | JASN Journal of the American Society of Nephrology | KIDNEY360 Accessing Our World From Every Angle | CJASN Clinical Journal of the American Society of Nephrology

ASN Journal Disclosure Form

As per ASN journal policy, I have disclosed any financial relationships or commitments I have held in the past 36 months as included below. I have listed my Current Employer below to indicate there is a relationship requiring disclosure. If no relationship exists, my Current Employer is not listed.

E. Akalin reports the following:
Employer: Montefiore Medical Center, Albert Einstein College of Medicine; Consultancy: Neurona; Research Funding: Caredx, NIH; and Honoraria: Otsuka, Apellis, Takeda.

I understand that the information above will be published within the journal article, if accepted, and that failure to comply and/or to accurately and completely report the potential financial conflicts of interest could lead to the following: 1) Prior to publication, article rejection, or 2) Post-publication, sanctions ranging from, but not limited to, issuing a correction, reporting the inaccurate information to the authors' institution, banning authors from submitting work to ASN journals for varying lengths of time, and/or retraction of the published work.

Name: Enver Akalin
Manuscript ID: JASN-2025-001600R1
Manuscript Title: Elevations in Donor-Derived Cell-Free DNA Predict Allograft Outcomes in Kidney Transplantation
Date of Completion: April 5, 2026
Disclosure Updated Date: April 5, 2026

ASN American Society of Nephrology | JASN Journal of the American Society of Nephrology | KIDNEY360 Accessing Our World From Every Angle | CJASN Clinical Journal of the American Society of Nephrology

ASN Journal Disclosure Form

As per ASN journal policy, I have disclosed any financial relationships or commitments I have held in the past 36 months as included below. I have listed my Current Employer below to indicate there is a relationship requiring disclosure. If no relationship exists, my Current Employer is not listed.

T. Alhamad reports the following:
Employer: Washington University in St. Louis; Consultancy: Veloxis ; Natera; CareDx;; Research Funding: CareDx; Angion; Natera; Europhines, Eldon, CSL Imagine, Allovir; Honoraria: CareDx; Veloxis; Sanofi, Anlym, Vertex; Advisory or Leadership Role: CareDx, Europhines, Horizon, Vertex; and Speakers Bureau: Sanofi.

I understand that the information above will be published within the journal article, if accepted, and that failure to comply and/or to accurately and completely report the potential financial conflicts of interest could lead to the following: 1) Prior to publication, article rejection, or 2) Post-publication, sanctions ranging from, but not limited to, issuing a correction, reporting the inaccurate information to the authors' institution, banning authors from submitting work to ASN journals for varying lengths of time, and/or retraction of the published work.

Name: Tarek Alhamad
Manuscript ID: JASN-2025-001600R2
Manuscript Title: Elevations in Donor-Derived Cell-Free DNA and Allograft Outcomes in Kidney Transplantation
Date of Completion: May 1, 2026
Disclosure Updated Date: May 1, 2026

ASN American Society of Nephrology | JASN Journal of the American Society of Nephrology | KIDNEY360 Accessing Our World From Every Angle | CJASN Clinical Journal of the American Society of Nephrology

ASN Journal Disclosure Form

As per ASN journal policy, I have disclosed any financial relationships or commitments I have held in the past 36 months as included below. I have listed my Current Employer below to indicate there is a relationship requiring disclosure. If no relationship exists, my Current Employer is not listed.

P. Anand reports the following:
Employer: Medical University of South Carolina; Consultancy: CareDx, Alexion, Verici, ThermoFischer; Ownership Interest: Stock - CareDx; Honoraria: Veloxis; Calliditas, Sanofi, Apellis; Speakers Bureau: Calliditas; Sanofi, Apellis; and Other Interests or Relationships: Board member of South Carolina Transplant Fund (Nonprofit).

I understand that the information above will be published within the journal article, if accepted, and that failure to comply and/or to accurately and completely report the potential financial conflicts of interest could lead to the following: 1) Prior to publication, article rejection, or 2) Post-publication, sanctions ranging from, but not limited to, issuing a correction, reporting the inaccurate information to the authors' institution, banning authors from submitting work to ASN journals for varying lengths of time, and/or retraction of the published work.

Name: Prince Mohan Anand
Manuscript ID: JASN-2025-001600R1
Manuscript Title: Elevations in Donor-Derived Cell-Free DNA Predict Allograft Outcomes in Kidney Transplantation
Date of Completion: April 2, 2026
Disclosure Updated Date: April 2, 2026

ASN American Society of Nephrology | JASN Journal of the American Society of Nephrology | KIDNEY360 Accessing Our World From Every Angle | CJASN Clinical Journal of the American Society of Nephrology

ASN Journal Disclosure Form

As per ASN journal policy, I have disclosed any financial relationships or commitments I have held in the past 36 months as included below. I have listed my Current Employer below to indicate there is a relationship requiring disclosure. If no relationship exists, my Current Employer is not listed.

S. Anand reports the following:
Employer: Fresenius Medical; Consultancy: CareDx; Research Funding: CareDX; Bayer; Alexion; Novartis; Honoraria: Alexion; CareDX; and Speakers Bureau: Alexion; CareDX ; Natera.

I understand that the information above will be published within the journal article, if accepted, and that failure to comply and/or to accurately and completely report the potential financial conflicts of interest could lead to the following: 1) Prior to publication, article rejection, or 2) Post-publication, sanctions ranging from, but not limited to, issuing a correction, reporting the inaccurate information to the authors' institution, banning authors from submitting work to ASN journals for varying lengths of time, and/or retraction of the published work.

Name: Sanjiv Anand
Manuscript ID: JASN-2025-001600R1
Manuscript Title: Elevations in Donor-Derived Cell-Free DNA Predict Allograft Outcomes in Kidney Transplantation
Date of Completion: April 22, 2026
Disclosure Updated Date: April 6, 2026

American Society of Nephrology
JASN Journal of the American Society of Nephrology
KIDNEY360 Accessing Our World From Every Angle
CJASN Clinical Journal of the American Society of Nephrology

ASN Journal Disclosure Form

As per ASN journal policy, I have disclosed any financial relationships or commitments I have held in the past 36 months as included below. I have listed my Current Employer below to indicate there is a relationship requiring disclosure. If no relationship exists, my Current Employer is not listed.

D. Brennan reports the following:
Employer: The Johns Hopkins University School of Medicine; Consultancy: CareDx, Calliditas, Medeor Therapeutics, Memo Therapeutics Natera, Travere, Sanofi, Vera Therapeutics; Research Funding: CareDx; and Advisory or Leadership Role: Editorial Board: Transplantation, UpToDate.

I understand that the information above will be published within the journal article, if accepted, and that failure to comply and/or to accurately and completely report the potential financial conflicts of interest could lead to the following: 1) Prior to publication, article rejection, or 2) Post-publication, sanctions ranging from, but not limited to, issuing a correction, reporting the inaccurate information to the authors' institution, banning authors from submitting work to ASN journals for varying lengths of time, and/or retraction of the published work.

Name: Daniel C. Brennan
Manuscript ID: JASN-2025-001600R1
Manuscript Title: Elevations in Donor-Derived Cell-Free DNA Predict Allograft Outcomes in Kidney Transplantation
Date of Completion: April 3, 2026
Disclosure Updated Date: April 3, 2026

ASN American Society of Nephrology | JASN Journal of the American Society of Nephrology | KIDNEY360 Accessing Our World From Every Angle | CJASN Clinical Journal of the American Society of Nephrology

ASN Journal Disclosure Form

As per ASN journal policy, I have disclosed any financial relationships or commitments I have held in the past 36 months as included below. I have listed my Current Employer below to indicate there is a relationship requiring disclosure. If no relationship exists, my Current Employer is not listed.

J. Bromberg reports the following:
Employer: University of Maryland; Research Funding: CareDx, Natera; and Patents or Royalties: University of Maryland.

I understand that the information above will be published within the journal article, if accepted, and that failure to comply and/or to accurately and completely report the potential financial conflicts of interest could lead to the following: 1) Prior to publication, article rejection, or 2) Post-publication, sanctions ranging from, but not limited to, issuing a correction, reporting the inaccurate information to the authors' institution, banning authors from submitting work to ASN journals for varying lengths of time, and/or retraction of the published work.

Name: Jonathan Bromberg
Manuscript ID: JASN-2025-001600R1
Manuscript Title: Elevations in Donor-Derived Cell-Free DNA Predict Allograft Outcomes in Kidney Transplantation
Date of Completion: April 10, 2026
Disclosure Updated Date: April 10, 2026

ASN American Society of Nephrology | JASN Journal of the American Society of Nephrology | KIDNEY360 Accessing Our World From Every Angle | CJASN Clinical Journal of the American Society of Nephrology

ASN Journal Disclosure Form

As per ASN journal policy, I have disclosed any financial relationships or commitments I have held in the past 36 months as included below. I have listed my Current Employer below to indicate there is a relationship requiring disclosure. If no relationship exists, my Current Employer is not listed.

S. Bunnapradist reports the following:
Employer: UCLA; Consultancy: CareDx, Eurofins, Merck, Natera, Nephrosant, Sanofi, Veloxis; Ownership Interest: None relevant; Research Funding: AlloVir, CareDx, CSL Behring, Eurofins, Merck, Natera, Sanofi; Honoraria: CareDx, Eurofins, Merck, Natera, Nephrosant, Sanofi, Takeda, Veloxis; Advisory or Leadership Role: CareDx, Eurofins, Merck, Natera, Nephrosant, Sanofi, Takeda, Veloxis, memo therapeutics, biogen; Speakers Bureau: CareDx, Eurofins, Natera, Sanofi, Takeda, Veloxis; and Other Interests or Relationships: Grant from OneLegacy.

I understand that the information above will be published within the journal article, if accepted, and that failure to comply and/or to accurately and completely report the potential financial conflicts of interest could lead to the following: 1) Prior to publication, article rejection, or 2) Post-publication, sanctions ranging from, but not limited to, issuing a correction, reporting the inaccurate information to the authors' institution, banning authors from submitting work to ASN journals for varying lengths of time, and/or retraction of the published work.

Name: Suphamai Bunnapradist
Manuscript ID: JASN-2025-001600R1
Manuscript Title: Elevations in Donor-Derived Cell-Free DNA Predict Allograft Outcomes in Kidney Transplantation
Date of Completion: April 13, 2026
Disclosure Updated Date: July 22, 2025

ASN
American Society of Nephrology
JASN
Journal of the American Society of Nephrology
KIDNEY360
Accessing Our World From Every Angle
CJASN
Clinical Journal of the American Society of Nephrology

ASN Journal Disclosure Form

As per ASN journal policy, I have disclosed any financial relationships or commitments I have held in the past 36 months as included below. I have listed my Current Employer below to indicate there is a relationship requiring disclosure. If no relationship exists, my Current Employer is not listed.

G. Burke reports the following:
Employer: University of Miami Miller School of Medicine; Consultancy: CareDx; Research Funding: Miami Transplant Institute; Honoraria: CareDx; and Advisory or Leadership Role: Associate Editor, Transplantation (Not paid).

I understand that the information above will be published within the journal article, if accepted, and that failure to comply and/or to accurately and completely report the potential financial conflicts of interest could lead to the following: 1) Prior to publication, article rejection, or 2) Post-publication, sanctions ranging from, but not limited to, issuing a correction, reporting the inaccurate information to the authors' institution, banning authors from submitting work to ASN journals for varying lengths of time, and/or retraction of the published work.

Name: George William Burke
Manuscript ID: JASN-/025-001600R1
Manuscript Title: Elevations in Donor-Derived Cell-Free DNA Predict Allograft Outcomes in Kidney Transplantation
Date of Completion: April 3, 2026
Disclosure Updated Date: April 3, 2026

ASN American Society of Nephrology | JASN Journal of the American Society of Nephrology | KIDNEY360 Accessing Our World From Every Angle | CJASN Clinical Journal of the American Society of Nephrology

ASN Journal Disclosure Form

As per ASN journal policy, I have disclosed any financial relationships or commitments I have held in the past 36 months as included below. I have listed my Current Employer below to indicate there is a relationship requiring disclosure. If no relationship exists, my Current Employer is not listed.

R. Choudhury reports the following:
Employer: University of Colorado Hospital

I understand that the information above will be published within the journal article, if accepted, and that failure to comply and/or to accurately and completely report the potential financial conflicts of interest could lead to the following: 1) Prior to publication, article rejection, or 2) Post-publication, sanctions ranging from, but not limited to, issuing a correction, reporting the inaccurate information to the authors' institution, banning authors from submitting work to ASN journals for varying lengths of time, and/or retraction of the published work.

Name: Rashikh A. Choudhury
Manuscript ID: JASN-2025-001600R2
Manuscript Title: Elevations in Donor-Derived Cell-Free DNA and Allograft Outcomes in Kidney Transplantation
Date of Completion: May 1, 2026
Disclosure Updated Date: May 1, 2026

ASN
American Society of Nephrology
JASN
Journal of the American Society of Nephrology
KIDNEY360
Accessing Our World From Every Angle
CJASN
Clinical Journal of the American Society of Nephrology

ASN Journal Disclosure Form

As per ASN journal policy, I have disclosed any financial relationships or commitments I have held in the past 36 months as included below. I have listed my Current Employer below to indicate there is a relationship requiring disclosure. If no relationship exists, my Current Employer is not listed.

P. Chuang reports the following:
Employer: CareDx; Ownership Interest: CareDx; Research Funding: CareDx; and Advisory or Leadership Role: CareDx.

I understand that the information above will be published within the journal article, if accepted, and that failure to comply and/or to accurately and completely report the potential financial conflicts of interest could lead to the following: 1) Prior to publication, article rejection, or 2) Post-publication, sanctions ranging from, but not limited to, issuing a correction, reporting the inaccurate information to the authors' institution, banning authors from submitting work to ASN journals for varying lengths of time, and/or retraction of the published work.

Name: Peale Chuang
Manuscript ID: JASN-2025-001600R1
Manuscript Title: Elevations in Donor-Derived Cell-Free DNA Predict Allograft Outcomes in Kidney Transplantation
Date of Completion: April 3, 2026
Disclosure Updated Date: April 3, 2026

ASN American Society of Nephrology | JASN Journal of the American Society of Nephrology | KIDNEY360 Accessing Our World From Every Angle | CJASN Clinical Journal of the American Society of Nephrology

ASN Journal Disclosure Form

As per ASN journal policy, I have disclosed any financial relationships or commitments I have held in the past 36 months as included below. I have listed my Current Employer below to indicate there is a relationship requiring disclosure. If no relationship exists, my Current Employer is not listed.

D. Cibrik reports the following:
Employer: University of Kansas Hospital; Consultancy: CareDx; Eledon; Biogen; Research Funding: PI on Bestow K207 multicenter study (Eledon) and PI on Transcend study (Biogen); Honoraria: Biogen; Eledon; CareDx; and Advisory or Leadership Role: Biogen steering commitee.

I understand that the information above will be published within the journal article, if accepted, and that failure to comply and/or to accurately and completely report the potential financial conflicts of interest could lead to the following: 1) Prior to publication, article rejection, or 2) Post-publication, sanctions ranging from, but not limited to, issuing a correction, reporting the inaccurate information to the authors' institution, banning authors from submitting work to ASN journals for varying lengths of time, and/or retraction of the published work.

Name: Diane Marie Cibrik
Manuscript ID: JASN-2025-001600R1
Manuscript Title: Elevations in Donor-Derived Cell-Free DNA Predict Allograft Outcomes in Kidney Transplantation
Date of Completion: April 10, 2026
Disclosure Updated Date: April 10, 2026

ASN Journal Disclosure Form

As per ASN journal policy, I have disclosed any financial relationships or commitments I have held in the past 36 months as included below. I have listed my Current Employer below to indicate there is a relationship requiring disclosure. If no relationship exists, my Current Employer is not listed.

M. Cooper reports the following:
Employer: Medical College of Wisconsin; Consultancy: CareDx; Natera; Specialist Direct; Advisory or Leadership Role: UNOS; National Kidney Foundation; National Kidney Registry; American Foundation for Donation and Transplant; International Pancreas and Islet Cell Transplant Association; Donate Life America;; and Speakers Bureau: Sanofi.

I understand that the information above will be published within the journal article, if accepted, and that failure to comply and/or to accurately and completely report the potential financial conflicts of interest could lead to the following: 1) Prior to publication, article rejection, or 2) Post-publication, sanctions ranging from, but not limited to, issuing a correction, reporting the inaccurate information to the authors' institution, banning authors from submitting work to ASN journals for varying lengths of time, and/or retraction of the published work.

Name: Matthew Cooper
Manuscript ID: JASN-2025-001600R1
Manuscript Title: Elevations in Donor-Derived Cell-Free DNA Predict Allograft Outcomes in Kidney Transplantation
Date of Completion: April 3, 2026
Disclosure Updated Date: August 25, 2025

ASN
American Society of Nephrology
JASN
Journal of the American Society of Nephrology
KIDNEY360
Accessing Our World From Every Angle
CJASN
Clinical Journal of the American Society of Nephrology

ASN Journal Disclosure Form

As per ASN journal policy, I have disclosed any financial relationships or commitments I have held in the past 36 months as included below. I have listed my Current Employer below to indicate there is a relationship requiring disclosure. If no relationship exists, my Current Employer is not listed.

N. Costa has nothing to disclose.

I understand that the information above will be published within the journal article, if accepted, and that failure to comply and/or to accurately and completely report the potential financial conflicts of interest could lead to the following: 1) Prior to publication, article rejection, or 2) Post-publication, sanctions ranging from, but not limited to, issuing a correction, reporting the inaccurate information to the authors' institution, banning authors from submitting work to ASN journals for varying lengths of time, and/or retraction of the published work.

Name: Nadiesda Costa
Manuscript ID: 2025-001600R1
Manuscript Title: Elevations in Donor-Derived Cell-Free DNA Predict Allograft Outcomes in Kidney Transplantation
Date of Completion: April 3, 2026
Disclosure Updated Date: March 13, 2026

ASN American Society of Nephrology JASN Journal of the American Society of Nephrology KIDNEY360 Accessing Our World From Every Angle CJASN Clinical Journal of the American Society of Nephrology

ASN Journal Disclosure Form

As per ASN journal policy, I have disclosed any financial relationships or commitments I have held in the past 36 months as included below. I have listed my Current Employer below to indicate there is a relationship requiring disclosure. If no relationship exists, my Current Employer is not listed.

C. Fan reports the following:
Employer: CareDx, Inc; Ownership Interest: CareDx Inc.; and Patents or Royalties: CareDx Inc.

I understand that the information above will be published within the journal article, if accepted, and that failure to comply and/or to accurately and completely report the potential financial conflicts of interest could lead to the following: 1) Prior to publication, article rejection, or 2) Post-publication, sanctions ranging from, but not limited to, issuing a correction, reporting the inaccurate information to the authors' institution, banning authors from submitting work to ASN journals for varying lengths of time, and/or retraction of the published work.

Name: Chun-Po Steve Fan
Manuscript ID: JASN-2025-001600R1
Manuscript Title: Elevations in Donor-Derived Cell-Free DNA Predict Allograft Outcomes in Kidney Transplantation
Date of Completion: April 3, 2026
Disclosure Updated Date: April 3, 2026

ASN
American Society of Nephrology
JASN
Journal of the American Society of Nephrology
KIDNEY360
Accessing Our World From Every Angle
CJASN
Clinical Journal of the American Society of Nephrology

ASN Journal Disclosure Form

As per ASN journal policy, I have disclosed any financial relationships or commitments I have held in the past 36 months as included below. I have listed my Current Employer below to indicate there is a relationship requiring disclosure. If no relationship exists, my Current Employer is not listed.

B. Fischbach reports the following:
Employer: Dallas Nephrology Associates; and Research Funding: Eli Lilly, Vertex, and ProKidney.

I understand that the information above will be published within the journal article, if accepted, and that failure to comply and/or to accurately and completely report the potential financial conflicts of interest could lead to the following: 1) Prior to publication, article rejection, or 2) Post-publication, sanctions ranging from, but not limited to, issuing a correction, reporting the inaccurate information to the authors' institution, banning authors from submitting work to ASN journals for varying lengths of time, and/or retraction of the published work.

Name: Bernard V. Fischbach
Manuscript ID: JASN-2025-001600R1
Manuscript Title: Elevations in Donor-Derived Cell-Free DNA Predict Allograft Outcomes in Kidney Transplantation
Date of Completion: April 3, 2026
Disclosure Updated Date: April 3, 2026

ASN American Society of Nephrology JASN Journal of the American Society of Nephrology KIDNEY360 Accessing Our World From Every Angle CJASN Clinical Journal of the American Society of Nephrology

ASN Journal Disclosure Form

As per ASN journal policy, I have disclosed any financial relationships or commitments I have held in the past 36 months as included below. I have listed my Current Employer below to indicate there is a relationship requiring disclosure. If no relationship exists, my Current Employer is not listed.

G. Guerra reports the following:
Employer: Jackson Memorial Hospital; 34 Lives; 1Elevan; Consultancy: 34 Lives; 1Elevan; and Advisory or Leadership Role: National Kidney Foundation - board member (not paid).

I understand that the information above will be published within the journal article, if accepted, and that failure to comply and/or to accurately and completely report the potential financial conflicts of interest could lead to the following: 1) Prior to publication, article rejection, or 2) Post-publication, sanctions ranging from, but not limited to, issuing a correction, reporting the inaccurate information to the authors' institution, banning authors from submitting work to ASN journals for varying lengths of time, and/or retraction of the published work.

Name: Giselle Guerra
Manuscript ID: Journal of American Society of Nephrology
Manuscript Title: "Elevations in Donor-Derived Cell-Free DNA and Allograft Outcomes in Kidney Transplantation."
Date of Completion: May 1, 2026
Disclosure Updated Date: May 1, 2026

ASN American Society of Nephrology | JASN Journal of the American Society of Nephrology | KIDNEY360 Accessing Our World From Every Angle | CJASN Clinical Journal of the American Society of Nephrology

ASN Journal Disclosure Form

As per ASN journal policy, I have disclosed any financial relationships or commitments I have held in the past 36 months as included below. I have listed my Current Employer below to indicate there is a relationship requiring disclosure. If no relationship exists, my Current Employer is not listed.

Y. Huang reports the following:
Employer: University of California, Davis

I understand that the information above will be published within the journal article, if accepted, and that failure to comply and/or to accurately and completely report the potential financial conflicts of interest could lead to the following: 1) Prior to publication, article rejection, or 2) Post-publication, sanctions ranging from, but not limited to, issuing a correction, reporting the inaccurate information to the authors' institution, banning authors from submitting work to ASN journals for varying lengths of time, and/or retraction of the published work.

Name: Yihung Huang
Manuscript ID: JASN-2025-001600R1
Manuscript Title: Elevations in Donor-Derived Cell-Free DNA Predict Allograft Outcomes in Kidney Transplantation
Date of Completion: April 6, 2026
Disclosure Updated Date: April 6, 2026

ASN
American Society of Nephrology
JASN
Journal of the American Society of Nephrology
KIDNEY360
Accessing Our World From Every Angle
CJASN
Clinical Journal of the American Society of Nephrology

ASN Journal Disclosure Form

As per ASN journal policy, I have disclosed any financial relationships or commitments I have held in the past 36 months as included below. I have listed my Current Employer below to indicate there is a relationship requiring disclosure. If no relationship exists, my Current Employer is not listed.

P. Jain reports the following:
Employer: Florida Kidney Physicians; Consultancy: Care Dx; ITB; Ownership Interest: Care Dx common stock; Honoraria: Care Dx;; ITB; and Speakers Bureau: ITB Med.

I understand that the information above will be published within the journal article, if accepted, and that failure to comply and/or to accurately and completely report the potential financial conflicts of interest could lead to the following: 1) Prior to publication, article rejection, or 2) Post-publication, sanctions ranging from, but not limited to, issuing a correction, reporting the inaccurate information to the authors' institution, banning authors from submitting work to ASN journals for varying lengths of time, and/or retraction of the published work.

Name: Pranjal Jain
Manuscript ID: JASN-2025-001600R1
Manuscript Title: Elevations in Donor-Derived Cell-Free DNA Predict Allograft Outcomes in Kidney Transplantation
Date of Completion: April 24, 2026
Disclosure Updated Date: April 24, 2026

ASN
American Society of Nephrology
JASN
Journal of the American Society of Nephrology
KIDNEY360
Accessing Our World From Every Angle
CJASN
Clinical Journal of the American Society of Nephrology

ASN Journal Disclosure Form

As per ASN journal policy, I have disclosed any financial relationships or commitments I have held in the past 36 months as included below. I have listed my Current Employer below to indicate there is a relationship requiring disclosure. If no relationship exists, my Current Employer is not listed.

M. Josephson reports the following:
Employer: University of Chicago; Consultancy: Vera Therapeutics; Alexion; and Advisory or Leadership Role: American Society of Nephrology; KDIGO.

I understand that the information above will be published within the journal article, if accepted, and that failure to comply and/or to accurately and completely report the potential financial conflicts of interest could lead to the following: 1) Prior to publication, article rejection, or 2) Post-publication, sanctions ranging from, but not limited to, issuing a correction, reporting the inaccurate information to the authors' institution, banning authors from submitting work to ASN journals for varying lengths of time, and/or retraction of the published work.

Name: Michelle A. Josephson
Manuscript ID: JASN-2025-001600R1
Manuscript Title: Elevations in Donor-Derived Cell-Free DNA Predict Allograft Outcomes in Kidney Transplantation
Date of Completion: April 8, 2026
Disclosure Updated Date: February 17, 2026

ASN
American Society of Nephrology
JASN
Journal of the American Society of Nephrology
KIDNEY360
Accessing Our World From Every Angle
CJASN
Clinical Journal of the American Society of Nephrology

ASN Journal Disclosure Form

As per ASN journal policy, I have disclosed any financial relationships or commitments I have held in the past 36 months as included below. I have listed my Current Employer below to indicate there is a relationship requiring disclosure. If no relationship exists, my Current Employer is not listed.

J. Klein reports the following:
Employer: University of Kansas; Research Funding: University of Kansas KOAR site PI, CareDx; University of Kansas OKRA site PI, CareDx; and Advisory or Leadership Role: KOAR manuscript steering committee member. Unpaid.

I understand that the information above will be published within the journal article, if accepted, and that failure to comply and/or to accurately and completely report the potential financial conflicts of interest could lead to the following: 1) Prior to publication, article rejection, or 2) Post-publication, sanctions ranging from, but not limited to, issuing a correction, reporting the inaccurate information to the authors' institution, banning authors from submitting work to ASN journals for varying lengths of time, and/or retraction of the published work.

Name: Jeffrey A. Klein
Manuscript ID: JASN-2025-001600R1
Manuscript Title: Elevations in Donor-Derived Cell-Free DNA Predict Allograft Outcomes in Kidney Transplantation
Date of Completion: April 3, 2026
Disclosure Updated Date: April 3, 2026

ASN American Society of Nephrology | JASN Journal of the American Society of Nephrology | KIDNEY360 Accessing Our World From Every Angle | CJASN Clinical Journal of the American Society of Nephrology

ASN Journal Disclosure Form

As per ASN journal policy, I have disclosed any financial relationships or commitments I have held in the past 36 months as included below. I have listed my Current Employer below to indicate there is a relationship requiring disclosure. If no relationship exists, my Current Employer is not listed.

S. Kulkarni reports the following:
Employer: Yale University (Active Employees & Faculty)

I understand that the information above will be published within the journal article, if accepted, and that failure to comply and/or to accurately and completely report the potential financial conflicts of interest could lead to the following: 1) Prior to publication, article rejection, or 2) Post-publication, sanctions ranging from, but not limited to, issuing a correction, reporting the inaccurate information to the authors' institution, banning authors from submitting work to ASN journals for varying lengths of time, and/or retraction of the published work.

Name: Sanjay Kulkarni
Manuscript ID: JASN-2025-001600R1
Manuscript Title: Elevations in Donor-Derived Cell-Free DNA Predict Allograft Outcomes in Kidney Transplantation
Date of Completion: April 28, 2026
Disclosure Updated Date: April 28, 2026

American Society of Nephrology | JASN Journal of the American Society of Nephrology | KIDNEY360 Accessing Our World From Every Angle | CJASN Clinical Journal of the American Society of Nephrology

ASN Journal Disclosure Form

As per ASN journal policy, I have disclosed any financial relationships or commitments I have held in the past 36 months as included below. I have listed my Current Employer below to indicate there is a relationship requiring disclosure. If no relationship exists, my Current Employer is not listed.

D. Kumar reports the following:
Employer: Virginia Commonwealth University Health System; and Speakers Bureau: CareDx; Themo Fisher.

I understand that the information above will be published within the journal article, if accepted, and that failure to comply and/or to accurately and completely report the potential financial conflicts of interest could lead to the following: 1) Prior to publication, article rejection, or 2) Post-publication, sanctions ranging from, but not limited to, issuing a correction, reporting the inaccurate information to the authors' institution, banning authors from submitting work to ASN journals for varying lengths of time, and/or retraction of the published work.

Name: Dhiren Kumar
Manuscript ID: JASN-2025-001600R1
Manuscript Title: Elevations in Donor-Derived Cell-Free DNA Predict Allograft Outcomes in Kidney Transplantation
Date of Completion: April 3, 2026
Disclosure Updated Date: April 3, 2026

ASN Journal Disclosure Form

As per ASN journal policy, I have disclosed any financial relationships or commitments I have held in the past 36 months as included below. I have listed my Current Employer below to indicate there is a relationship requiring disclosure. If no relationship exists, my Current Employer is not listed.

E. Langewisch reports the following:
Employer: University of Nebraska Medical Center; and Research Funding: Principle Investigator in Clinical Trials with following sponsors; Biogen; CSL Behring; argenx BV; CareDx.

I understand that the information above will be published within the journal article, if accepted, and that failure to comply and/or to accurately and completely report the potential financial conflicts of interest could lead to the following: 1) Prior to publication, article rejection, or 2) Post-publication, sanctions ranging from, but not limited to, issuing a correction, reporting the inaccurate information to the authors' institution, banning authors from submitting work to ASN journals for varying lengths of time, and/or retraction of the published work.

Name: Eric D. Langewisch
Manuscript ID: JASN-2025-001600R1
Manuscript Title: Elevations in Donor-Derived Cell-Free DNA Predict Allograft Outcomes in Kidney Transplantation
Date of Completion: April 6, 2026
Disclosure Updated Date: April 6, 2026

ASN American Society of Nephrology | JASN Journal of the American Society of Nephrology | KIDNEY360 Accessing Our World From Every Angle | CJASN Clinical Journal of the American Society of Nephrology

ASN Journal Disclosure Form

As per ASN journal policy, I have disclosed any financial relationships or commitments I have held in the past 36 months as included below. I have listed my Current Employer below to indicate there is a relationship requiring disclosure. If no relationship exists, my Current Employer is not listed.

A. Langone reports the following:
Research Funding: Natera; Veloxis

I understand that the information above will be published within the journal article, if accepted, and that failure to comply and/or to accurately and completely report the potential financial conflicts of interest could lead to the following: 1) Prior to publication, article rejection, or 2) Post-publication, sanctions ranging from, but not limited to, issuing a correction, reporting the inaccurate information to the authors' institution, banning authors from submitting work to ASN journals for varying lengths of time, and/or retraction of the published work.

Name: Anthony J. Langone
Manuscript ID: JASN-2025-001600R1
Manuscript Title: Elevations in Donor-Derived Cell-Free DNA Predict Allograft Outcomes in Kidney Transplantation
Date of Completion: April 10, 2026
Disclosure Updated Date: April 10, 2026

ASN
American Society of Nephrology
JASN
Journal of the American Society of Nephrology
KIDNEY360
Accessing Our World From Every Angle
CJASN
Clinical Journal of the American Society of Nephrology

ASN Journal Disclosure Form

As per ASN journal policy, I have disclosed any financial relationships or commitments I have held in the past 36 months as included below. I have listed my Current Employer below to indicate there is a relationship requiring disclosure. If no relationship exists, my Current Employer is not listed.

N. Leca reports the following:
Employer: University of Washington; Consultancy: Alexion, Natera, Novartis, Travere, Sanofi, Veloxis; and Research Funding: Novartis CareDx Natera Transplant Genomics Verici MEMO Hansa Alexion Biogen ArgenX.

I understand that the information above will be published within the journal article, if accepted, and that failure to comply and/or to accurately and completely report the potential financial conflicts of interest could lead to the following: 1) Prior to publication, article rejection, or 2) Post-publication, sanctions ranging from, but not limited to, issuing a correction, reporting the inaccurate information to the authors' institution, banning authors from submitting work to ASN journals for varying lengths of time, and/or retraction of the published work.

Name: Nicolae Leca
Manuscript ID: JASN-2025-001600R1
Manuscript Title: Elevations in Donor-Derived Cell-Free DNA Predict Allograft Outcomes in Kidney Transplantation
Date of Completion: April 3, 2026
Disclosure Updated Date: December 2, 2025

ASN
American Society of Nephrology
JASN
Journal of the American Society of Nephrology
KIDNEY360
Accessing Our World From Every Angle
CJASN
Clinical Journal of the American Society of Nephrology

ASN Journal Disclosure Form

As per ASN journal policy, I have disclosed any financial relationships or commitments I have held in the past 36 months as included below. I have listed my Current Employer below to indicate there is a relationship requiring disclosure. If no relationship exists, my Current Employer is not listed.

D. Leeser reports the following:
Employer: Brody School of Medicine at East Carolina University; and Research Funding: Natera, Veloxis, CareDx, Eurofin.

I understand that the information above will be published within the journal article, if accepted, and that failure to comply and/or to accurately and completely report the potential financial conflicts of interest could lead to the following: 1) Prior to publication, article rejection, or 2) Post-publication, sanctions ranging from, but not limited to, issuing a correction, reporting the inaccurate information to the authors' institution, banning authors from submitting work to ASN journals for varying lengths of time, and/or retraction of the published work.

Name: David Black Leeser
Manuscript ID: JASN-2025-001600R1
Manuscript Title: Elevations in Donor-Derived Cell-Free DNA Predict Allograft Outcomes in Kidney Transplantation
Date of Completion: April 6, 2026
Disclosure Updated Date: April 6, 2026

ASN American Society of Nephrology | JASN Journal of the American Society of Nephrology | KIDNEY360 Accessing Our World From Every Angle | CJASN Clinical Journal of the American Society of Nephrology

ASN Journal Disclosure Form

As per ASN journal policy, I have disclosed any financial relationships or commitments I have held in the past 36 months as included below. I have listed my Current Employer below to indicate there is a relationship requiring disclosure. If no relationship exists, my Current Employer is not listed.

Z. Lu reports the following:
Employer: CareDx; Hologic; and Ownership Interest: CareDx.

I understand that the information above will be published within the journal article, if accepted, and that failure to comply and/or to accurately and completely report the potential financial conflicts of interest could lead to the following: 1) Prior to publication, article rejection, or 2) Post-publication, sanctions ranging from, but not limited to, issuing a correction, reporting the inaccurate information to the authors' institution, banning authors from submitting work to ASN journals for varying lengths of time, and/or retraction of the published work.

Name: Zhenqiang Lu
Manuscript ID: JASN-2025-001600R1
Manuscript Title: Elevations in Donor-Derived Cell-Free DNA Predict Allograft Outcomes in Kidney Transplantation
Date of Completion: April 3, 2026
Disclosure Updated Date: April 3, 2026

ASN American Society of Nephrology
JASN Journal of the American Society of Nephrology
KIDNEY360 Accessing Our World From Every Angle
CJASN Clinical Journal of the American Society of Nephrology

ASN Journal Disclosure Form

As per ASN journal policy, I have disclosed any financial relationships or commitments I have held in the past 36 months as included below. I have listed my Current Employer below to indicate there is a relationship requiring disclosure. If no relationship exists, my Current Employer is not listed.

D. Mandelbrot reports the following:
Employer: UWHealth (Relationship has not ended); Consultancy: Natera (Relationship has not ended); Thermo-Fisher (Relationship has not ended); and Research Funding: CareDx (Relationship has not ended).

I understand that the information above will be published within the journal article, if accepted, and that failure to comply and/or to accurately and completely report the potential financial conflicts of interest could lead to the following: 1) Prior to publication, article rejection, or 2) Post-publication, sanctions ranging from, but not limited to, issuing a correction, reporting the inaccurate information to the authors' institution, banning authors from submitting work to ASN journals for varying lengths of time, and/or retraction of the published work.

Name: Didier A. Mandelbrot
Manuscript ID: JASN-2025-001600R1
Manuscript Title: Elevations in Donor-Derived Cell-Free DNA Predict Allograft Outcomes in Kidney Transplantation
Date of Completion: April 29, 2026
Disclosure Updated Date: April 29, 2026

ASN American Society of Nephrology
JASN Journal of the American Society of Nephrology
KIDNEY360 Accessing Our World From Every Angle
CJASN Clinical Journal of the American Society of Nephrology

ASN Journal Disclosure Form

As per ASN journal policy, I have disclosed any financial relationships or commitments I have held in the past 36 months as included below. I have listed my Current Employer below to indicate there is a relationship requiring disclosure. If no relationship exists, my Current Employer is not listed.

T. Maw reports the following:
Employer: Keck School of Medicine USC

I understand that the information above will be published within the journal article, if accepted, and that failure to comply and/or to accurately and completely report the potential financial conflicts of interest could lead to the following: 1) Prior to publication, article rejection, or 2) Post-publication, sanctions ranging from, but not limited to, issuing a correction, reporting the inaccurate information to the authors' institution, banning authors from submitting work to ASN journals for varying lengths of time, and/or retraction of the published work.

Name: Thin Thin Maw
Manuscript ID: JASN-2025-001600R1
Manuscript Title: Elevations in Donor-Derived Cell-Free DNA Predict Allograft Outcomes in Kidney Transplantation
Date of Completion: April 11, 2026
Disclosure Updated Date: April 11, 2026

ASN American Society of Nephrology | JASN Journal of the American Society of Nephrology | KIDNEY360 Accessing Our World From Every Angle | CJASN Clinical Journal of the American Society of Nephrology

ASN Journal Disclosure Form

As per ASN journal policy, I have disclosed any financial relationships or commitments I have held in the past 36 months as included below. I have listed my Current Employer below to indicate there is a relationship requiring disclosure. If no relationship exists, my Current Employer is not listed.

M. McGrath reports the following:
Employer: Brigham and Women's Hospital; VA Boston Medical Center; Consultancy: Me: Alexion Pharmaceuticals, Vera Therapeutics, Astra Zeneca; Spouse: GSK and Vydus; Research Funding: Self: Lexicon Pharmaceuticals; Spouse: Lexicon Pharmaceuticals, Novartis, Satellite Healthcare, Fifth Eye, NIDDK; and Other Interests or Relationships: Self: Editorial Director, nephSAP.

I understand that the information above will be published within the journal article, if accepted, and that failure to comply and/or to accurately and completely report the potential financial conflicts of interest could lead to the following: 1) Prior to publication, article rejection, or 2) Post-publication, sanctions ranging from, but not limited to, issuing a correction, reporting the inaccurate information to the authors’ institution, banning authors from submitting work to ASN journals for varying lengths of time, and/or retraction of the published work.

Name: Martina M. McGrath
Manuscript ID: JASN-2025-001600R1
Manuscript Title: Elevations in Donor-Derived Cell-Free DNA Predict Allograft Outcomes in Kidney Transplantation
Date of Completion: April 13, 2026
Disclosure Updated Date: April 13, 2026

ASN American Society of Nephrology | JASN Journal of the American Society of Nephrology | KIDNEY360 Accessing Our World From Every Angle | CJASN Clinical Journal of the American Society of Nephrology

ASN Journal Disclosure Form

As per ASN journal policy, I have disclosed any financial relationships or commitments I have held in the past 36 months as included below. I have listed my Current Employer below to indicate there is a relationship requiring disclosure. If no relationship exists, my Current Employer is not listed.

M. Merzkani has nothing to disclose.

I understand that the information above will be published within the journal article, if accepted, and that failure to comply and/or to accurately and completely report the potential financial conflicts of interest could lead to the following: 1) Prior to publication, article rejection, or 2) Post-publication, sanctions ranging from, but not limited to, issuing a correction, reporting the inaccurate information to the authors' institution, banning authors from submitting work to ASN journals for varying lengths of time, and/or retraction of the published work.

Name: Massini Merzkani
Manuscript ID: JASN-2025-001600R1
Manuscript Title: Elevations in Donor-Derived Cell-Free DNA Predict Allograft Outcomes in Kidney Transplantation
Date of Completion: April 22, 2026
Disclosure Updated Date: April 10, 2026

ASN American Society of Nephrology | JASN Journal of the American Society of Nephrology | KIDNEY360 Accessing Our World From Every Angle | CJASN Clinical Journal of the American Society of Nephrology

ASN Journal Disclosure Form

As per ASN journal policy, I have disclosed any financial relationships or commitments I have held in the past 36 months as included below. I have listed my Current Employer below to indicate there is a relationship requiring disclosure. If no relationship exists, my Current Employer is not listed.

M. Narayanan reports the following:
Employer: Baylor Scott & White Healthcare - not for profit; Consultancy: Novartis, Otsuka; Research Funding: GSK; Novartis; Amgen; Care Dx, Vertex, Aurinia; and Speakers Bureau: Veloxis, Boehringer Ingelheim, Travere.

I understand that the information above will be published within the journal article, if accepted, and that failure to comply and/or to accurately and completely report the potential financial conflicts of interest could lead to the following: 1) Prior to publication, article rejection, or 2) Post-publication, sanctions ranging from, but not limited to, issuing a correction, reporting the inaccurate information to the authors' institution, banning authors from submitting work to ASN journals for varying lengths of time, and/or retraction of the published work.

Name: Mohanram Narayanan
Manuscript ID: JASN-2025-001600R1
Manuscript Title: Elevations in Donor-Derived Cell-Free DNA Predict Allograft Outcomes in Kidney Transplantation
Date of Completion: April 3, 2026
Disclosure Updated Date: April 3, 2026

ASN American Society of Nephrology | JASN Journal of the American Society of Nephrology | KIDNEY360 Accessing Our World From Every Angle | CJASN Clinical Journal of the American Society of Nephrology

ASN Journal Disclosure Form

As per ASN journal policy, I have disclosed any financial relationships or commitments I have held in the past 36 months as included below. I have listed my Current Employer below to indicate there is a relationship requiring disclosure. If no relationship exists, my Current Employer is not listed.

O. Olaitan reports the following:
Employer: Rush University Medical Center; Research Funding: CareDx; and Honoraria: CareDx.

I understand that the information above will be published within the journal article, if accepted, and that failure to comply and/or to accurately and completely report the potential financial conflicts of interest could lead to the following: 1) Prior to publication, article rejection, or 2) Post-publication, sanctions ranging from, but not limited to, issuing a correction, reporting the inaccurate information to the authors' institution, banning authors from submitting work to ASN journals for varying lengths of time, and/or retraction of the published work.

Name: Oyedolamu Olaitan
Manuscript ID: JASN-2025-001600R1
Manuscript Title: Elevations in Donor-Derived Cell-Free DNA Predict Allograft Outcomes in Kidney Transplantation,
Date of Completion: April 4, 2026
Disclosure Updated Date: April 4, 2026

ASN American Society of Nephrology | JASN Journal of the American Society of Nephrology | KIDNEY360 Accessing Our World From Every Angle | CJASN Clinical Journal of the American Society of Nephrology

ASN Journal Disclosure Form

As per ASN journal policy, I have disclosed any financial relationships or commitments I have held in the past 36 months as included below. I have listed my Current Employer below to indicate there is a relationship requiring disclosure. If no relationship exists, my Current Employer is not listed.

A. Paramesh reports the following:
Employer: Tulane University School of Medicine; Consultancy: Specialists Direct; Monogram Health; ThermoFisher Sciences One Lambda; Ownership Interest: Multiple stocks (>100) held in a professionally managed brokerage account; Honoraria: ThermoFisher Sciences One Lambda; and Advisory or Leadership Role: Louisiana Organ Procurement Agency; National Kidney Foundation Louisiana.

I understand that the information above will be published within the journal article, if accepted, and that failure to comply and/or to accurately and completely report the potential financial conflicts of interest could lead to the following: 1) Prior to publication, article rejection, or 2) Post-publication, sanctions ranging from, but not limited to, issuing a correction, reporting the inaccurate information to the authors' institution, banning authors from submitting work to ASN journals for varying lengths of time, and/or retraction of the published work.

Name: Anil S. Paramesh
Manuscript ID: JASN-2025-001600R2
Manuscript Title: Elevations in Donor-Derived Cell-Free DNA and Allograft Outcomes in Kidney Transplantation
Date of Completion: May 2, 2026
Disclosure Updated Date: May 1, 2026

ASN American Society of Nephrology | JASN Journal of the American Society of Nephrology | KIDNEY360 Accessing Our World From Every Angle | CJASN Clinical Journal of the American Society of Nephrology

ASN Journal Disclosure Form

As per ASN journal policy, I have disclosed any financial relationships or commitments I have held in the past 36 months as included below. I have listed my Current Employer below to indicate there is a relationship requiring disclosure. If no relationship exists, my Current Employer is not listed.

A. Patel reports the following:
Employer: Cooperman Barnabas Medical Center; and Speakers Bureau: Merck.

I understand that the information above will be published within the journal article, if accepted, and that failure to comply and/or to accurately and completely report the potential financial conflicts of interest could lead to the following: 1) Prior to publication, article rejection, or 2) Post-publication, sanctions ranging from, but not limited to, issuing a correction, reporting the inaccurate information to the authors' institution, banning authors from submitting work to ASN journals for varying lengths of time, and/or retraction of the published work.

Name: Anup M. Patel
Manuscript ID: JASN-2025-001600R1
Manuscript Title: Elevations in Donor-Derived Cell-Free DNA Predict Allograft Outcomes in Kidney Transplantation
Date of Completion: April 6, 2026
Disclosure Updated Date: April 6, 2026

ASN American Society of Nephrology | JASN Journal of the American Society of Nephrology | KIDNEY360 Accessing Our World From Every Angle | CJASN Clinical Journal of the American Society of Nephrology

ASN Journal Disclosure Form

As per ASN journal policy, I have disclosed any financial relationships or commitments I have held in the past 36 months as included below. I have listed my Current Employer below to indicate there is a relationship requiring disclosure. If no relationship exists, my Current Employer is not listed.

N. Permpalung reports the following:
Employer: Johns Hopkins University; Consultancy: ClearView; Research Funding: CareDx, IMMY Diagnostics, Merck, Scynexis, Pearl Diagnostics, FujiFilm, Applied BioCode, Zepto Life Technology;; Advisory or Leadership Role: Basilea (DSMB Chair); Pulmocide (DRC);; and Other Interests or Relationships: Medical Mycology (Associate Editor); Journal of Heart and Lung Transplantation Open (Editorial Board); Member of the IDSA Guideline Committee for Aspergillosis ;.

I understand that the information above will be published within the journal article, if accepted, and that failure to comply and/or to accurately and completely report the potential financial conflicts of interest could lead to the following: 1) Prior to publication, article rejection, or 2) Post-publication, sanctions ranging from, but not limited to, issuing a correction, reporting the inaccurate information to the authors' institution, banning authors from submitting work to ASN journals for varying lengths of time, and/or retraction of the published work.

Name: Nitipong Permpalung
Manuscript ID: JASN-2025-001600R1
Manuscript Title: Elevations in Donor-Derived Cell-Free DNA Predict Allograft Outcomes in Kidney Transplantation
Date of Completion: April 3, 2026
Disclosure Updated Date: April 3, 2026

ASN American Society of Nephrology JASN Journal of the American Society of Nephrology KIDNEY360 Accessing Our World From Every Angle CJASN Clinical Journal of the American Society of Nephrology

ASN Journal Disclosure Form

As per ASN journal policy, I have disclosed any financial relationships or commitments I have held in the past 36 months as included below. I have listed my Current Employer below to indicate there is a relationship requiring disclosure. If no relationship exists, my Current Employer is not listed.

T. Pesavento reports the following:
Employer: Ohio State University; Katherine M Cyran Breast Center; Ownership Interest: Katherine M Cyran Breast Center; Research Funding: NIH; Talaris; CareDx; Natera, Biogen, Argenx, Memo Therapeutics; and Advisory or Leadership Role: Ohio Solid Organ Transplant Consortium; Board of Directors;   OPTN committee member.

I understand that the information above will be published within the journal article, if accepted, and that failure to comply and/or to accurately and completely report the potential financial conflicts of interest could lead to the following: 1) Prior to publication, article rejection, or 2) Post-publication, sanctions ranging from, but not limited to, issuing a correction, reporting the inaccurate information to the authors' institution, banning authors from submitting work to ASN journals for varying lengths of time, and/or retraction of the published work.

Name: Todd E. Pesavento
Manuscript ID: JASN-2025-001600R1
Manuscript Title: Elevations in Donor-Derived Cell-Free DNA Predict Allograft Outcomes in Kidney Transplantation
Date of Completion: April 13, 2026
Disclosure Updated Date: April 13, 2026

ASN American Society of Nephrology
JASN Journal of the American Society of Nephrology
KIDNEY360 Accessing Our World From Every Angle
CJASN Clinical Journal of the American Society of Nephrology

ASN Journal Disclosure Form

As per ASN journal policy, I have disclosed any financial relationships or commitments I have held in the past 36 months as included below. I have listed my Current Employer below to indicate there is a relationship requiring disclosure. If no relationship exists, my Current Employer is not listed.

E. Poggio reports the following:
Employer: Cleveland Clinic; Consultancy: CareDx, Transplant Genomics, Natera, Horizon, ThermoFisher, Veloxis, Sanofi; and Honoraria: CareDx, Transplant Genomics, Natera, Horizon, ThermoFisher, Veloxis, Sanofi.

I understand that the information above will be published within the journal article, if accepted, and that failure to comply and/or to accurately and completely report the potential financial conflicts of interest could lead to the following: 1) Prior to publication, article rejection, or 2) Post-publication, sanctions ranging from, but not limited to, issuing a correction, reporting the inaccurate information to the authors' institution, banning authors from submitting work to ASN journals for varying lengths of time, and/or retraction of the published work.

Name: Emilio D. Poggio
Manuscript ID: JASN-2025-001600R2
Manuscript Title: Elevations in Donor-Derived Cell-Free DNA and Allograft Outcomes in Kidney Transplantation
Date of Completion: May 1, 2026
Disclosure Updated Date: February 19, 2026

ASN American Society of Nephrology JASN Journal of the American Society of Nephrology KIDNEY360 Accessing Our World From Every Angle CJASN Clinical Journal of the American Society of Nephrology

ASN Journal Disclosure Form

As per ASN journal policy, I have disclosed any financial relationships or commitments I have held in the past 36 months as included below. I have listed my Current Employer below to indicate there is a relationship requiring disclosure. If no relationship exists, my Current Employer is not listed.

R. Ponsirenas reports the following:
Employer: CareDx; and Honoraria: CareDx, Salary.

I understand that the information above will be published within the journal article, if accepted, and that failure to comply and/or to accurately and completely report the potential financial conflicts of interest could lead to the following: 1) Prior to publication, article rejection, or 2) Post-publication, sanctions ranging from, but not limited to, issuing a correction, reporting the inaccurate information to the authors' institution, banning authors from submitting work to ASN journals for varying lengths of time, and/or retraction of the published work.

Name: Renata Ponsirenas
Manuscript ID: JASN-2025-001600R1
Manuscript Title: Elevations in Donor-Derived Cell-Free DNA Predict Allograft Outcomes in Kidney Transplantation
Date of Completion: April 3, 2026
Disclosure Updated Date: April 3, 2026

ASN American Society of Nephrology | JASN Journal of the American Society of Nephrology | KIDNEY360 Accessing Our World From Every Angle | CJASN Clinical Journal of the American Society of Nephrology

ASN Journal Disclosure Form

As per ASN journal policy, I have disclosed any financial relationships or commitments I have held in the past 36 months as included below. I have listed my Current Employer below to indicate there is a relationship requiring disclosure. If no relationship exists, my Current Employer is not listed.

A. Qannus reports the following:
Employer: Banner University of Arizona Hospital

I understand that the information above will be published within the journal article, if accepted, and that failure to comply and/or to accurately and completely report the potential financial conflicts of interest could lead to the following: 1) Prior to publication, article rejection, or 2) Post-publication, sanctions ranging from, but not limited to, issuing a correction, reporting the inaccurate information to the authors' institution, banning authors from submitting work to ASN journals for varying lengths of time, and/or retraction of the published work.

Name: Abd Assalam Qannus
Manuscript ID: JASN-2025-001600
Manuscript Title: Elevations in Donor Derived Cell-Free DNA Predict Allograft Outcomes in Kidney Transplantation
Date of Completion: April 15, 2026
Disclosure Updated Date: April 15, 2026

ASN Journal Disclosure Form

As per ASN journal policy, I have disclosed any financial relationships or commitments I have held in the past 36 months as included below. I have listed my Current Employer below to indicate there is a relationship requiring disclosure. If no relationship exists, my Current Employer is not listed.

L. Shen reports the following:
Employer: CareDx; Oracle; and Ownership Interest: CareDx; Oracle.

I understand that the information above will be published within the journal article, if accepted, and that failure to comply and/or to accurately and completely report the potential financial conflicts of interest could lead to the following: 1) Prior to publication, article rejection, or 2) Post-publication, sanctions ranging from, but not limited to, issuing a correction, reporting the inaccurate information to the authors' institution, banning authors from submitting work to ASN journals for varying lengths of time, and/or retraction of the published work.

Name: Ling Shen
Manuscript ID: JASN-2025-001600R1)
Manuscript Title: Elevations in Donor-Derived Cell-Free DNA Predict Allograft Outcomes in Kidney Transplantation
Date of Completion: April 3, 2026
Disclosure Updated Date: April 3, 2026

ASN
American Society of Nephrology
JASN
Journal of the American Society of Nephrology
KIDNEY360
Accessing Our World From Every Angle
CJASN
Clinical Journal of the American Society of Nephrology

ASN Journal Disclosure Form

As per ASN journal policy, I have disclosed any financial relationships or commitments I have held in the past 36 months as included below. I have listed my Current Employer below to indicate there is a relationship requiring disclosure. If no relationship exists, my Current Employer is not listed.

F. Shihab reports the following:
Employer: University of Utah Health Science Center; Consultancy: Biogen; Research Funding: CareDx; Natera; Eurofins; Advisory or Leadership Role: American Foundation of Donation and Transplantation, Clinical Transplantation; and Other Interests or Relationships: AST, NKF, AFDT.

I understand that the information above will be published within the journal article, if accepted, and that failure to comply and/or to accurately and completely report the potential financial conflicts of interest could lead to the following: 1) Prior to publication, article rejection, or 2) Post-publication, sanctions ranging from, but not limited to, issuing a correction, reporting the inaccurate information to the authors' institution, banning authors from submitting work to ASN journals for varying lengths of time, and/or retraction of the published work.

Name: Fuad Shihab
Manuscript ID: JASN-2025-001600R1
Manuscript Title: Elevations in Donor-Derived Cell-Free DNA Predict Allograft Outcomes in Kidney Transplantation
Date of Completion: April 2, 2026
Disclosure Updated Date: April 2, 2026

ASN American Society of Nephrology
JASN Journal of the American Society of Nephrology
KIDNEY360 Accessing Our World From Every Angle
CJASN Clinical Journal of the American Society of Nephrology

ASN Journal Disclosure Form

As per ASN journal policy, I have disclosed any financial relationships or commitments I have held in the past 36 months as included below. I have listed my Current Employer below to indicate there is a relationship requiring disclosure. If no relationship exists, my Current Employer is not listed.

K. Sureshkumar reports the following:
Ownership Interest: Apple, Microsoft, Alphabet, Meta, Amazon, Invidia, Tesla, Disney, Boeing, Alibaba, Snowflake, Walmart, Pfizer, Moderna, J&J, AirBnB, Uber, Marathon oil, Juniper network, Advanced micro devices, Plug powers, Expedia. Berkshire Hathaway B, Palantir; Research Funding: CareDx; Honoraria: Spherix global insights; Health Advances; Market Plus, DeciBio; Jupiter Life Science Consulting LLC; and Advisory or Leadership Role: Medical Advisory Board, Center for Organ Recovery and Education, no payments received.

I understand that the information above will be published within the journal article, if accepted, and that failure to comply and/or to accurately and completely report the potential financial conflicts of interest could lead to the following: 1) Prior to publication, article rejection, or 2) Post-publication, sanctions ranging from, but not limited to, issuing a correction, reporting the inaccurate information to the authors’ institution, banning authors from submitting work to ASN journals for varying lengths of time, and/or retraction of the published work.

Name: Kalathil K. Sureshkumar
Manuscript ID: JASN-2025-001600R1
Manuscript Title: Elevations in Donor-Derived Cell-Free DNA Predict Allograft Outcomes in Kidney Transplantation
Date of Completion: April 11, 2026
Disclosure Updated Date: April 11, 2026

ASN American Society of Nephrology
JASN Journal of the American Society of Nephrology
KIDNEY360 Accessing Our World From Every Angle
CJASN Clinical Journal of the American Society of Nephrology

ASN Journal Disclosure Form

As per ASN journal policy, I have disclosed any financial relationships or commitments I have held in the past 36 months as included below. I have listed my Current Employer below to indicate there is a relationship requiring disclosure. If no relationship exists, my Current Employer is not listed.

D. Taber reports the following:
Employer: Medical University of South Carolina; Consultancy: Veloxis, Sanofi; Research Funding: Veloxis, CareDx, Merck, Takeda, Thermo Fisher; and Advisory or Leadership Role: American Society of Transplantation.

I understand that the information above will be published within the journal article, if accepted, and that failure to comply and/or to accurately and completely report the potential financial conflicts of interest could lead to the following: 1) Prior to publication, article rejection, or 2) Post-publication, sanctions ranging from, but not limited to, issuing a correction, reporting the inaccurate information to the authors' institution, banning authors from submitting work to ASN journals for varying lengths of time, and/or retraction of the published work.

Name: David J. Taber
Manuscript ID: JASN-2025-001600R1
Manuscript Title: Elevations in Donor-Derived Cell-Free DNA Predict Allograft Outcomes in Kidney Transplantation
Date of Completion: April 10, 2026
Disclosure Updated Date: February 5, 2026

ASN
American Society of Nephrology
JASN
Journal of the American Society of Nephrology
KIDNEY360
Accessing Our World From Every Angle
CJASN
Clinical Journal of the American Society of Nephrology

ASN Journal Disclosure Form

As per ASN journal policy, I have disclosed any financial relationships or commitments I have held in the past 36 months as included below. I have listed my Current Employer below to indicate there is a relationship requiring disclosure. If no relationship exists, my Current Employer is not listed.

B. Tanriover reports the following:
Employer: University of Arizona College of Medicine-Tucson; Research Funding: Veloxis Pharmaceuticals; Honoraria: ThermoFisher / Advisory Board ; Calliditas / Advisory Board ; Veloxis / Advisory Board; and Advisory or Leadership Role: ThermoFisher Scientific ; Calliditas Therapeutics; Veloxis Pharmaceuticals.

I understand that the information above will be published within the journal article, if accepted, and that failure to comply and/or to accurately and completely report the potential financial conflicts of interest could lead to the following: 1) Prior to publication, article rejection, or 2) Post-publication, sanctions ranging from, but not limited to, issuing a correction, reporting the inaccurate information to the authors' institution, banning authors from submitting work to ASN journals for varying lengths of time, and/or retraction of the published work.

Name: Bekir Tanriover
Manuscript ID: JASN-2025-001600R1
Manuscript Title: Elevations in Donor-Derived Cell-Free DNA Predict Allograft Outcomes in Kidney Transplantation
Date of Completion: April 3, 2026
Disclosure Updated Date: April 3, 2026

ASN American Society of Nephrology | JASN Journal of the American Society of Nephrology | KIDNEY360 Accessing Our World From Every Angle | CJASN Clinical Journal of the American Society of Nephrology

ASN Journal Disclosure Form

As per ASN journal policy, I have disclosed any financial relationships or commitments I have held in the past 36 months as included below. I have listed my Current Employer below to indicate there is a relationship requiring disclosure. If no relationship exists, my Current Employer is not listed.

I. Tawhari reports the following:
Employer: King Khalid University College of Medicine, Department of Internal Medicine, Division of Nephrology; University of Illinois Chicago, Department of Medicine, Division of Nephrology

I understand that the information above will be published within the journal article, if accepted, and that failure to comply and/or to accurately and completely report the potential financial conflicts of interest could lead to the following: 1) Prior to publication, article rejection, or 2) Post-publication, sanctions ranging from, but not limited to, issuing a correction, reporting the inaccurate information to the authors' institution, banning authors from submitting work to ASN journals for varying lengths of time, and/or retraction of the published work.

Name: Ibrahim Tawhari
Manuscript ID: JASN-2025-001600R2
Manuscript Title: Elevations in Donor-Derived Cell-Free DNA and Allograft Outcomes in Kidney Transplantation
Date of Completion: May 1, 2026
Disclosure Updated Date: April 3, 2026

ASN
American Society of Nephrology
JASN
Journal of the American Society of Nephrology
KIDNEY360
Accessing Our World From Every Angle
CJASN
Clinical Journal of the American Society of Nephrology

ASN Journal Disclosure Form

As per ASN journal policy, I have disclosed any financial relationships or commitments I have held in the past 36 months as included below. I have listed my Current Employer below to indicate there is a relationship requiring disclosure. If no relationship exists, my Current Employer is not listed.

P. Vaitla has nothing to disclose.

I understand that the information above will be published within the journal article, if accepted, and that failure to comply and/or to accurately and completely report the potential financial conflicts of interest could lead to the following: 1) Prior to publication, article rejection, or 2) Post-publication, sanctions ranging from, but not limited to, issuing a correction, reporting the inaccurate information to the authors' institution, banning authors from submitting work to ASN journals for varying lengths of time, and/or retraction of the published work.

Name: Pradeep Vaitla
Manuscript ID: JASN-2025-001600R1
Manuscript Title: Elevations in Donor-Derived Cell-Free DNA Predict Allograft Outcomes in Kidney Transplantation
Date of Completion: April 10, 2026
Disclosure Updated Date: April 10, 2026

ASN American Society of Nephrology | JASN Journal of the American Society of Nephrology | KIDNEY360 Accessing Our World From Every Angle | CJASN Clinical Journal of the American Society of Nephrology

ASN Journal Disclosure Form

As per ASN journal policy, I have disclosed any financial relationships or commitments I have held in the past 36 months as included below. I have listed my Current Employer below to indicate there is a relationship requiring disclosure. If no relationship exists, my Current Employer is not listed.

M. Weir reports the following:
Employer: University of Maryland School of Medicine; Consultancy: AstraZeneca, Bayer, Novo Nordisk, Vera, Mineralys, CSL Vifor, Medtronic, Intercept, Daxor , Corcept, Boehringer-Ingelheim; and Honoraria: Same as above for ad hoc advisory board meetings.

I understand that the information above will be published within the journal article, if accepted, and that failure to comply and/or to accurately and completely report the potential financial conflicts of interest could lead to the following: 1) Prior to publication, article rejection, or 2) Post-publication, sanctions ranging from, but not limited to, issuing a correction, reporting the inaccurate information to the authors' institution, banning authors from submitting work to ASN journals for varying lengths of time, and/or retraction of the published work.

Name: Matthew R. Weir
Manuscript ID: JASN-2025-001600R1
Manuscript Title: Elevations of donor-derived cell free DNA predict allograftoutcomes in kidney transplantation
Date of Completion: April 3, 2026
Disclosure Updated Date: January 14, 2026

ASN
American Society of Nephrology
JASN
Journal of the American Society of Nephrology
KIDNEY360
Accessing Our World From Every Angle
CJASN
Clinical Journal of the American Society of Nephrology

ASN Journal Disclosure Form

As per ASN journal policy, I have disclosed any financial relationships or commitments I have held in the past 36 months as included below. I have listed my Current Employer below to indicate there is a relationship requiring disclosure. If no relationship exists, my Current Employer is not listed.

A. Wiseman reports the following:
Consultancy: Invivyd (Relationship ended on 3/6/2026); Veloxis (Relationship ended on 12/1/2025); Biogen (Relationship ended on 11/10/2025); Sanofi (Relationship has not ended); Alexion (Relationship has not ended); Transplant Genomics/Eurofins (Relationship ended on 1/1/2026); and Speakers Bureau: Sanofi (Relationship has not ended); Takeda (Relationship ended on 6/1/2025).

I understand that the information above will be published within the journal article, if accepted, and that failure to comply and/or to accurately and completely report the potential financial conflicts of interest could lead to the following: 1) Prior to publication, article rejection, or 2) Post-publication, sanctions ranging from, but not limited to, issuing a correction, reporting the inaccurate information to the authors' institution, banning authors from submitting work to ASN journals for varying lengths of time, and/or retraction of the published work.

Name: Alexander C. Wiseman
Manuscript ID: JASN-2025-001600R1
Manuscript Title: Elevations in Donor-Derived Cell-Free DNA Predict Allograft Outcomes in Kidney Transplantation
Date of Completion: April 30, 2026
Disclosure Updated Date: March 6, 2026

ASN Journal Disclosure Form

As per ASN journal policy, I have disclosed any financial relationships or commitments I have held in the past 36 months as included below. I have listed my Current Employer below to indicate there is a relationship requiring disclosure. If no relationship exists, my Current Employer is not listed.

D. Wojciechowski reports the following:
Consultancy: Memo Therapeutics (Relationship has not ended); Natera (Relationship has not ended); Argenx (Relationship has not ended); and Research Funding: Memo Therapeutics (Relationship has not ended); Biogen (Relationship has not ended); Alexion (Relationship has not ended); CareDx (Relationship has not ended); Natera (Relationship has not ended); Argenx (Relationship has not ended).

I understand that the information above will be published within the journal article, if accepted, and that failure to comply and/or to accurately and completely report the potential financial conflicts of interest could lead to the following: 1) Prior to publication, article rejection, or 2) Post-publication, sanctions ranging from, but not limited to, issuing a correction, reporting the inaccurate information to the authors' institution, banning authors from submitting work to ASN journals for varying lengths of time, and/or retraction of the published work.

Name: David Wojciechowski
Manuscript ID: JASN-2025-001600R2
Manuscript Title: Elevations in Donor-Derived Cell-Free DNA and Allograft Outcomes in Kidney Transplantation
Date of Completion: May 4, 2026
Disclosure Updated Date: April 3, 2026

ASN American Society of Nephrology | JASN Journal of the American Society of Nephrology | KIDNEY360 Accessing Our World From Every Angle | CJASN Clinical Journal of the American Society of Nephrology

ASN Journal Disclosure Form

As per ASN journal policy, I have disclosed any financial relationships or commitments I have held in the past 36 months as included below. I have listed my Current Employer below to indicate there is a relationship requiring disclosure. If no relationship exists, my Current Employer is not listed.

R. Woodward reports the following:
Employer: CareDx; and Ownership Interest: CareDx.

I understand that the information above will be published within the journal article, if accepted, and that failure to comply and/or to accurately and completely report the potential financial conflicts of interest could lead to the following: 1) Prior to publication, article rejection, or 2) Post-publication, sanctions ranging from, but not limited to, issuing a correction, reporting the inaccurate information to the authors' institution, banning authors from submitting work to ASN journals for varying lengths of time, and/or retraction of the published work.

Name: Robert Woodward
Manuscript ID: JASN-2025-001600R1
Manuscript Title: Elevations in Donor-Derived Cell-Free DNA Predict Allograft Outcomes in Kidney Transplantation
Date of Completion: April 4, 2026
Disclosure Updated Date: March 17, 2026

ASN Journal Disclosure Form

As per ASN journal policy, I have disclosed any financial relationships or commitments I have held in the past 36 months as included below. I have listed my Current Employer below to indicate there is a relationship requiring disclosure. If no relationship exists, my Current Employer is not listed.

Z. Zaky reports the following:
Employer: Cleveland Clinic

I understand that the information above will be published within the journal article, if accepted, and that failure to comply and/or to accurately and completely report the potential financial conflicts of interest could lead to the following: 1) Prior to publication, article rejection, or 2) Post-publication, sanctions ranging from, but not limited to, issuing a correction, reporting the inaccurate information to the authors' institution, banning authors from submitting work to ASN journals for varying lengths of time, and/or retraction of the published work.

Name: Ziad S. Zaky
Manuscript ID: JASN-2025-001600R1
Manuscript Title: Elevations in Donor-Derived Cell-Free DNA Predict Allograft Outcomes in Kidney Transplantation
Date of Completion: April 3, 2026
Disclosure Updated Date: April 3, 2026
