# Supplementary material for: Elevations in Donor-Derived Cell-Free DNA and Allograft Outcomes in Kidney Transplantation
Source: J Am Soc Nephrol. 2026 Jun 16;37(11):2517–28. doi: 10.1681/ASN.0000001133 (PMC13742986; doi:10.1681/ASN.0000001133)
Supplement: Supplementary file 2 [file jasn-37-2517-s002.pdf]

Supplemental Material

Elevations in Donor-Derived Cell-Free DNA and Allograft Outcomes in Kidney Transplantation

Table of Contents

Supplemental Section 1. Patient Post-Transplant Trajectory 1

Supplemental Figure 1. Representative Patient Trajectories According to dd-cfDNA State 1

Supplemental Section 2. KOAR Registry Cohort Is Representative of the Contemporary United States Transplant Cohort 2

Supplemental Table 1. Comparison of Baseline Characteristics Between KOAR Study Cohort and National SRTR Kidney Transplant Recipients (2018–2020) 2

Supplemental Section 3. Biopsy Results 3

Supplemental Table 2. Biopsy Results According to First Biopsy 3

Supplemental Table 3. Histopathologic Findings Among Biopsies Classified as No Rejection, by dd-cfDNA Group 4

Supplemental Section 4. Allograft Dysfunction 5

Supplemental Section 4.1. eGFR Trajectory Analysis 5

Supplemental Figure 2. Longitudinal eGFR Trajectories by dd-cfDNA Group and CKD Stage Over 36 Months 5

Supplemental Section 4.2. Allograft Dysfunction in Patients with Complete eGFR Data Through Year 3 5

Supplemental Figure 3. Allograft Dysfunction by dd-cfDNA Group in Patients with Complete eGFR Data Through Year 3 5

Supplemental Section 5. Sensitivity Analyses for Allograft Loss 7

Supplemental Table 4. Cause-Specific Hazard Regression with dd-cfDNA as a Time-Varying, Continuous Covariate 7

Supplemental Section 6. Landmark Analyses 8

Supplemental Table 5. Landmark Analysis to Estimate the Hazard Ratio of 3-Year Allograft Loss at 3-, 6-, 12-, and 18-Month Post-Transplant 8

Supplemental Section 7. Causes of Allograft Loss 9

Supplemental Table 6. Causes of Allograft Loss by dd-cfDNA Group 9

Supplemental 1. Patient Post-Transplant Trajectory

Patients start in the Low dd-cfDNA state after transplantation. Upon the first dd-cfDNA elevation, they may transition from Low to either the Intermediate or High state, remaining in that state for the remainder of the study. Patients may subsequently transition only to the terminal states of allograft loss or death.

To illustrate these transitions, representative individual trajectories were plotted from the multistate model. Each panel depicts a kidney transplant recipient followed longitudinally after transplant.

These trajectories illustrate key transition patterns observed in the multistate model: patients may remain stable in the Low dd-cfDNA state, experience transient elevations followed by return to Low, or progress to and persist in Intermediate or High states over time, reflecting the dynamic nature of graft injury and recovery.

Supplemental Figure 1. Representative Patient Trajectories According to dd-cfDNA State

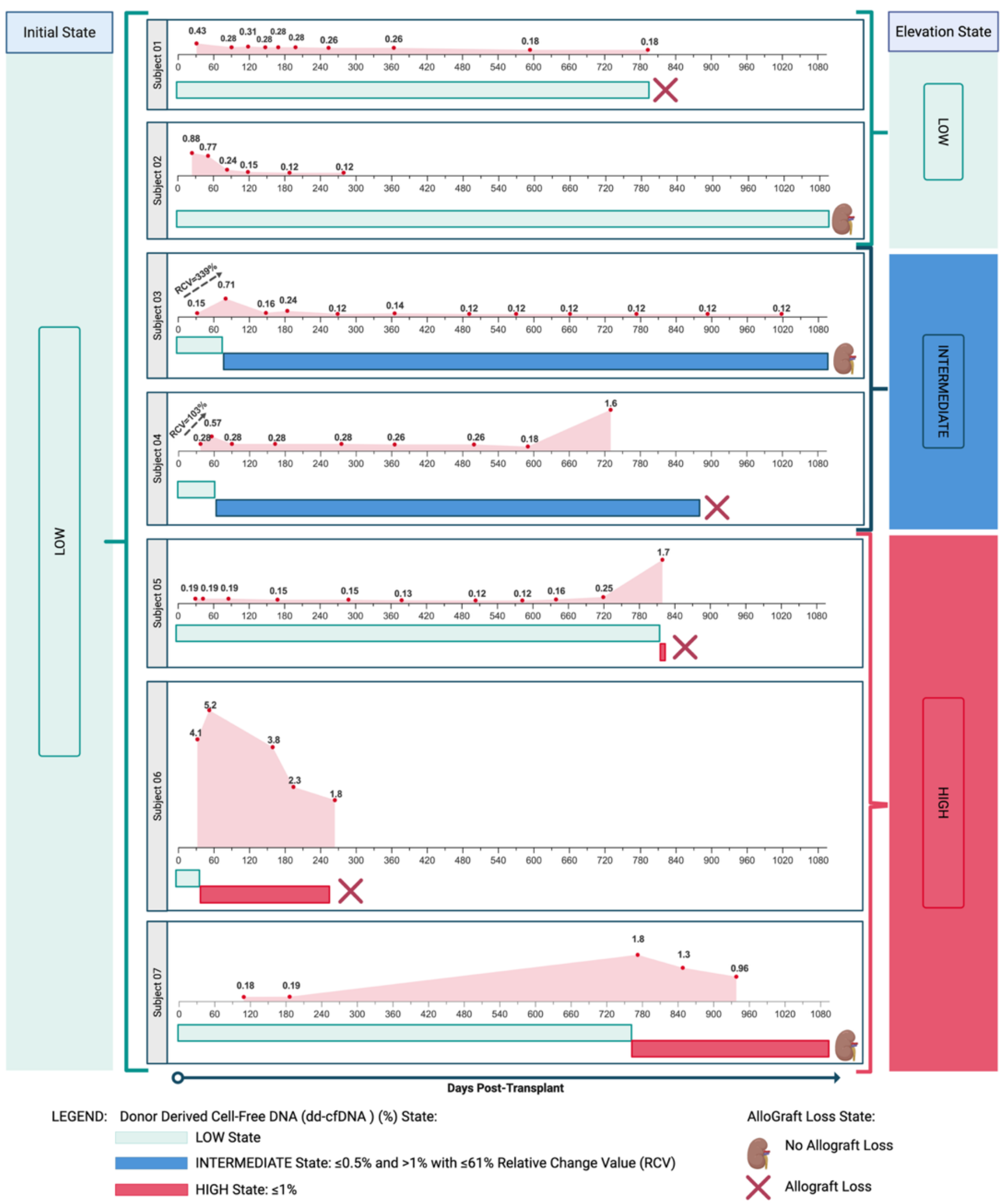

Supplemental Figure 1. *Each panel depicts a representative kidney transplant recipient followed longitudinally after transplant. Green bars indicate periods in the Low dd-cfDNA state (no elevation), blue bars indicate the Intermediate dd-cfDNA state (≥0.5% and <1% with ≥61% RCV), and red bars indicate the High dd-cfDNA state (≥1%). A red "X" represents allograft loss, and the kidney icon*

indicates graft survival at last follow-up. The trajectories illustrate how patients transition from Low to Intermediate or High dd-cfDNA states over time, demonstrating the time-dependent dd-cfDNA trajectory patterns modeled by the multistate model.

Supplemental 2. KOAR Registry Cohort Is Representative of Its Contemporary United States Transplant Cohort

The Kidney Allograft Outcomes AlloSure® Registry (KOAR) registry (NCT03326076) enrolled 1,743 kidney transplant recipients with transplants performed between January 2018 and December 2020 at 36 U.S. transplant centers. The study cohort of 1,258 patients was compared against the Scientific Registry of Transplant Recipients (SRTR) national cohort to confirm demographic representativeness.

Supplemental Table 1. Comparison of Baseline Characteristics Between KOAR Study Cohort and National SRTR Kidney Transplant Recipients (2018–2020)

Characteristic	KOAR (N = 1,258)	SRTR (N = 53,062)
**Age, mean ± SD**	54.5 ± 13.2	53.4 ± 13.5
**Age, n (%)**		
19–29	66 (5)	3,105 (6)
30–39	157 (13)	6,410 (12)
40–49	202 (16)	9,610 (18)
50–59	303 (24)	13,591 (26)
60–69	418 (33)	14,815 (28)
≥70	112 (9)	5,531 (10)
**Sex, n (%)**		
Male	780 (62)	32,256 (61)
Female	478 (38)	20,806 (39)
**Race, n (%)**		
White	656 (52)	33,706 (64)
Black or African American	392 (31)	14,430 (27)
Asian	78 (6)	3,779 (7)
Other	102 (8)	1,147 (2)
Unknown	30 (2)	0
**Prior Transplant, n (%)**		
No	1,119 (89)	46,587 (88)
Yes	139 (11)	6,475 (12)
**Donor Type, n (%)**		
Living	275 (22)	18,223 (34)
Deceased	983 (78)	34,839 (66)

KOAR — Kidney Allograft Outcomes AlloSure Registry; SRTR — Scientific Registry of Transplant Recipients; SD — Standard Deviation.

Supplemental 3. Biopsy Results

Summary of the first biopsy results after dd-cfDNA elevation in patients who transitioned to the Intermediate or High groups and the first biopsy performed (if any) in patients who remained in the Low group. A majority of patients in the Low group did not undergo biopsy (63.4%), compared to 69.9% in the Intermediate and 40.3% in the High group. Among those with a biopsy, the median time from transplant to biopsy in the Low group was 98 days. The median time from dd-cfDNA elevation to biopsy was 97 days in the Intermediate group and 25 days in the High group.

Rejection was more frequently identified in biopsies performed after dd-cfDNA elevation. Biopsy-proven rejection occurred in 4.0% of Low group patients, 17.1% of Intermediate, and 26.6% of High group patients. The dominant phenotype in the Low and Intermediate groups was T-cell mediated rejection (TCMR), with no ABMR reported in the Low group. In contrast, nearly half of rejection episodes in the High group were ABMR or mixed phenotypes, indicating a more severe histological pattern associated with higher dd-cfDNA levels.

Among biopsies classified as No Rejection, histopathologic findings were further characterized using the Banff Classification. Of 368 No Rejection biopsies, 187 (51%) had abnormal findings. Twenty-two biopsies demonstrated microvascular inflammation not meeting Banff 2019 ABMR criteria (Banff Category 2), predominantly in the High group (17%). Fifty-two biopsies had interstitial fibrosis and tubular atrophy as the sole finding (Banff Category 5), with higher IFTA grades observed in the Intermediate group. The remaining 80 biopsies had Category 6 diagnoses, with acute tubular injury being the most common finding across all groups.

Supplemental Table 2. Biopsy Results According with First Biopsy*

	Low (N = 809)	Intermediate (N = 216)	High (N = 233)
**Biopsy Results***			
No Biopsy n(%)	513 (63)	151 (70)	94 (40)
Days to 1st biopsy post-transplant, Median (IQR)	98 (42, 188)		
Days to 1st biopsy from dd-cfDNA elevation, Median (IQR)		97 (37, 265)	25 (13, 102)
**Biopsy Diagnosis n(%)**			
No Rejection	255 (86)	37 (57)	76 (55)
Borderline**	24 (8)	8 (12)	13 (9)
TCMR Only	16 (5)	15 (23)	26 (19)
ABMR Only	0 (0)	4 (6)	16 (12)
Mixed	1 (<1)	1 (2)	8 (6)

** Results reflect the first biopsy after dd-cfDNA elevation in patients who transitioned to the Intermediate or High groups. For Low group patients, biopsy results reflect the first biopsy if performed.*

*** Borderline was not considered rejection*

TCMR — T-cell Mediated Rejection; ABMR — Antibody Mediated Rejection; IQR — Interquartile Range.

Supplemental Table 3. Histopathologic Findings Among Biopsies Classified as No Rejection, by dd-cfDNA Group

No Rejection biopsies	Low N=255	Intermediate N=37	High (N=76)
Without abnormal findings†, n(%)	157 (62)	16 (43)	41 (54)
With abnormal findings, n(%)	98 (38)	21 (57)	35 (46)
**Histopathologic Finding**	**Low N=98**	**Intermediate N=21**	**High N=35**
**Banff Category 2: Antibody-Mediated Changes†† (N=22)**			
MVI, DSA-negative, C4d-negative, n(%)	5 (5.1)	4 (19.0)	13 (37.1)
**Banff Category 5: Interstitial Fibrosis and Tubular Atrophy§ (N=52)**			
IFTA Grade I (ct1 or ci1), n(%)	34 (34.7)	4 (19.0)	4 (11.4)
IFTA Grade II (ct2 or ci2)¶, n(%)	3 (3.1)	3 (14.3)	1 (2.9)
IFTA Grade III (ct3 or ci3), n(%)	1 (1.0)	1 (4.8)	1 (2.9)
**Banff Category 6: Other Changes Not Caused by Rejection‖ (N=80)**			
Acute Tubular Injury / Necrosis, n	27	5	9
Isometric Tubular Epithelial Vacuolization, n	2	1	1
Polyomavirus Nephropathy (BK/PVN), n	4	1	3
Pyelonephritis, n	2	0	0
Calcineurin Inhibitor Toxicity*, n	2	0	1
Focal Segmental Glomerulosclerosis, n	6	0	0
IgA Nephropathy, n	2	2	0
Membranous Nephropathy, n	1	0	0
Lupus Nephritis, n	1	0	0
Other Glomerulopathy‡, n	13	1	3

† Includes biopsies with no specific histopathologic finding (Banff Category 1: ct0/ci0 or missing scores).

†† Banff Category 2 per the Banff 2022 Classification. Biopsies with microvascular inflammation (g+ptc ≥2) not meeting Banff 2019 criteria for antibody-mediated rejection (absence of donor-specific antibodies and/or C4d).

§ Banff Category 5 (IFTA) grading among 52 patients with interstitial fibrosis and/or tubular atrophy as the sole abnormal finding: Grade I = ct1 or ci1 (mild); Grade II = ct2 or ci2 (moderate); Grade III = ct3 or ci3 (severe). ct — tubular atrophy; ci — interstitial fibrosis.

¶ One patient (Low group) had cg1b (GBM double contours ≤25% of capillary loops) with DSA-negative status. This finding does not meet Banff 2019 or 2022 criteria for ABMR in the absence of DSA, MVI, or C4d. The patient is classified under Category 5 Grade II based on concurrent ct2, ci2.

‖ Category 6 includes 80 unique patients. Individual diagnoses are not mutually exclusive; patients may have had concurrent findings (e.g., ATI with CNI toxicity or glomerulopathy). Counts reflect the number of patients with each diagnosis; percentages are not reported to avoid overestimation due to overlapping diagnoses.

** CNI Toxicity overlaps with ATI/ATN: 3 patients had ATI with concurrent CNI toxicity (Low: 2; High: 1).*

‡ Includes crescentic GN and unspecified glomerulopathy.

ATI — Acute Tubular Injury; ATN — Acute Tubular Necrosis; MVI — Microvascular Inflammation; ABMR — Antibody-Mediated Rejection; DSA — Donor-Specific Antibodies; ITEV — Isometric Tubular Epithelial Vacuolization; IFTA — Interstitial Fibrosis and Tubular Atrophy; PVN — Polyomavirus Nephropathy; CNI — Calcineurin Inhibitor; FSGS — Focal Segmental Glomerulosclerosis; GN — Glomerulonephritis.

Supplemental 4. Allograft Dysfunction

Supplemental 4.1 eGFR Trajectory Analysis

To evaluate the distribution and temporal evolution of kidney function across dd-cfDNA trajectory groups, longitudinal estimated glomerular filtration rate (eGFR) values were analyzed monthly over the 36-month follow-up period. Patients were stratified into three dd-cfDNA trajectory groups (Low, Intermediate, and High) based on their first qualifying dd-cfDNA elevation, and eGFR values were categorized according to chronic kidney disease (CKD) stages: ≥60 mL/min/1.73 m² (CKD G1–G2), 45–59 mL/min/1.73 m² (CKD G3a), 30–44 mL/min/1.73 m² (CKD G3b), and ≤30 mL/min/1.73 m² (CKD G4–G5).

At the time of dd-cfDNA elevation, 42.7% of patients in the Intermediate group and 33.0% in the High group maintained preserved renal function (eGFR ≥ 60 mL/min/1.73 m²). Over follow-up, eGFR trajectories diverged progressively across groups, with the proportion of patients exhibiting eGFR ≤ 30 mL/min/1.73 m² increasing stepwise from 3.7–7.5% in the Low group to 7.4–12.8% in the Intermediate group and 9.2–20.0% in the High group (p<0.001).

Supplemental Figure 2. Longitudinal eGFR Trajectories by dd-cfDNA Group and CKD Stage Over 36 Months

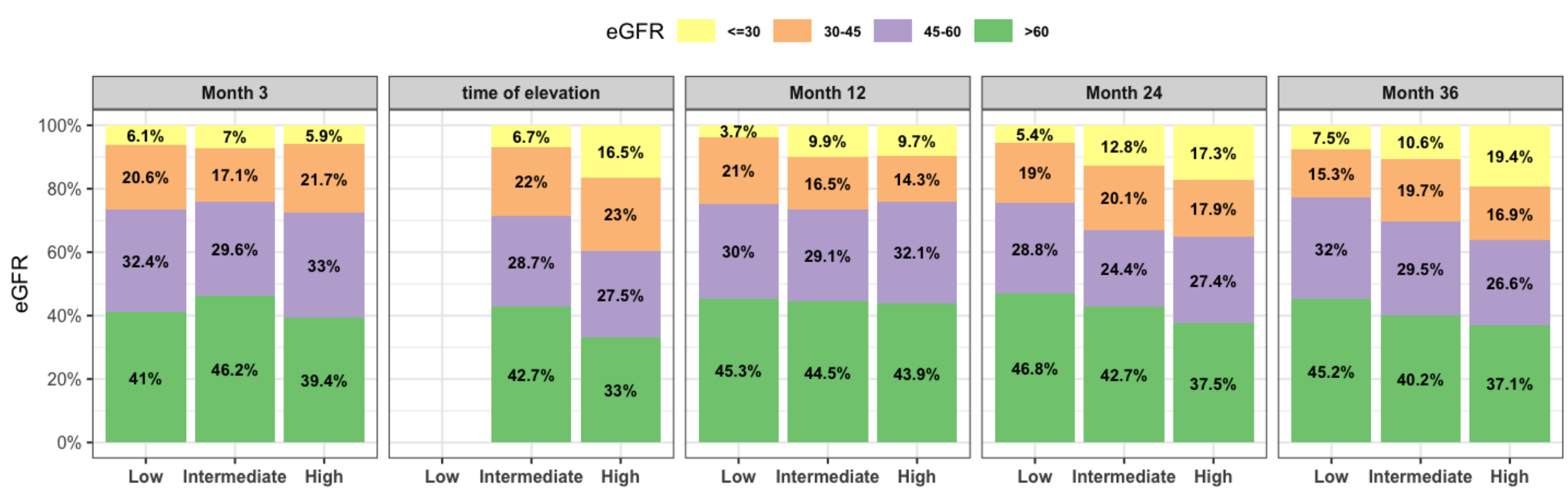

Supplemental Figure 2. *Longitudinal eGFR (mL/min/1.73 m²) by dd-cfDNA group (Low, Intermediate, High) and CKD stage (G1–G5), illustrating preserved renal function at dd-cfDNA elevation and progressive divergence over 36 months. Time of elevation refers to eGFR at time of dd-cfDNA elevation. dd-cfDNA, donor-derived cell-free DNA; eGFR, estimated glomerular filtration rate; CKD, chronic kidney disease.*

Supplemental 4.2 Allograft Dysfunction in Patients with Complete eGFR Data Through Year 3

To assess whether eGFR data availability at later time points influenced the primary allograft dysfunction findings, we performed an analysis restricted to patients with complete eGFR data available at both Month 12 and Month 36. Among 599 patients with complete longitudinal eGFR data (Low N=375, Intermediate N=115, High N=109), the pattern of allograft dysfunction was consistent with the primary analysis. At Month 3, rates were comparable across groups (Low 12.6%, Intermediate 12.6%, High 12.3%). By Month 12, dysfunction diverged substantially (Low 6.4%, Intermediate 15.7%, High 17.4%) and continued to increase through Month 36 (Low 8.5%, Intermediate 12.2%, High 22.0%; p<0.001), consistent with the findings in the full cohort.

Supplemental Figure 3. Allograft Dysfunction by dd-cfDNA Group in Patients with Complete eGFR Data Through Year 3

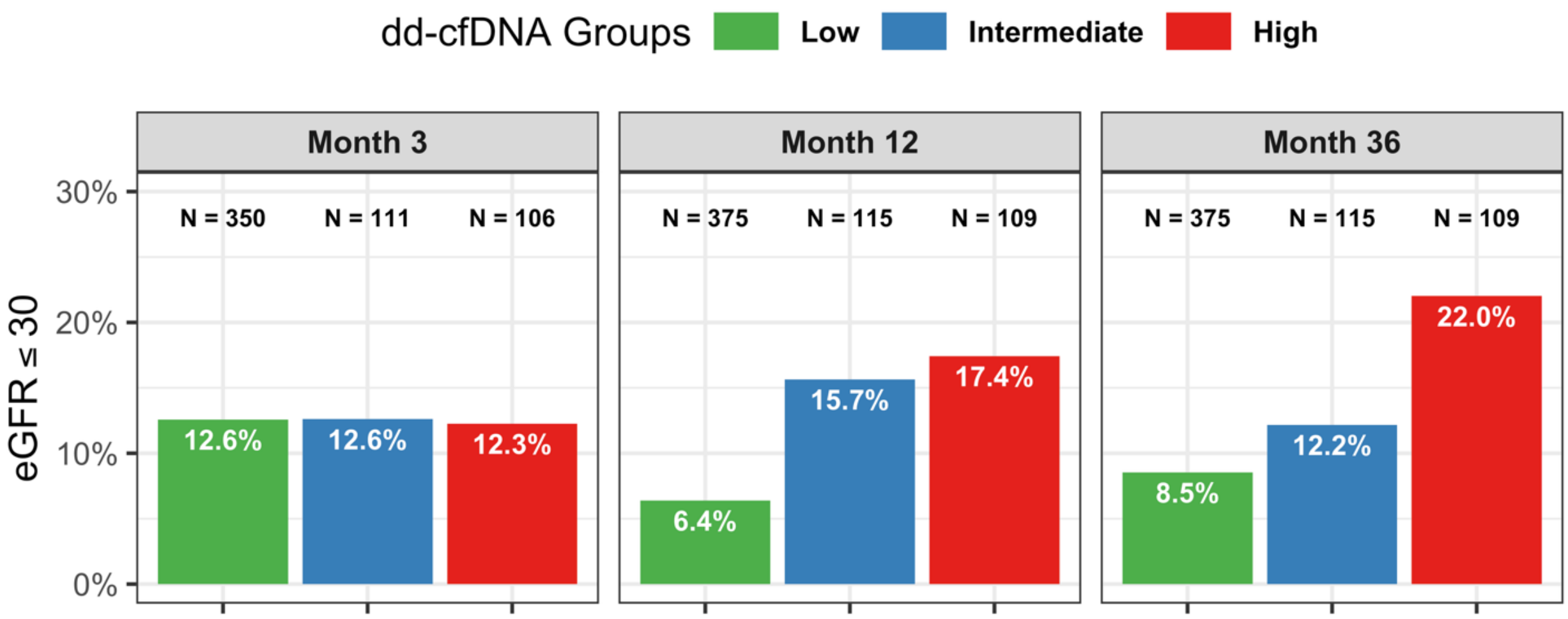

Supplemental Figure 3. *Proportion of patients with eGFR ≤30 mL/min/1.73 m² at Months 3, 12, and 36, restricted to patients with complete eGFR data at Months 12 and 36 (Low N=375, Intermediate N=115, High N=109). The divergence in allograft dysfunction across dd-cfDNA groups was consistent with the primary analysis. dd-cfDNA, donor-derived cell-free DNA; eGFR, estimated glomerular filtration rate.*

Supplemental 5. Sensitivity Analyses for Allograft Loss

This sensitivity analysis modeled dd-cfDNA as a time-varying continuous covariate using cause-specific hazard regression. The results support the findings in the main analysis - higher values were significantly associated with allograft loss.

Supplemental Table 4. Cause-specific Hazard Regression with dd-cfDNA as a time-varying, continuous covariate

	Cause specific HR	95% CI	P-value
**Unadjusted**			
dd-cfDNA (per % increase)	1.21	[1.09, 1.35]	<0.001
**Covariate adjusted**			
dd-cfDNA (per % increase)	1.16	[1.02, 1.33]	0.02
cPRA ≥80%	1.36	[0.52, 3.56]	0.53
Prior transplant	3.44	[1.32, 8.94]	0.01

** Death was modeled as a competing risk.*

HR — Hazard Ratio; dd-cfDNA — Donor-Derived Cell-Free DNA; cPRA — calculated Panel Reactive Antibodies.

Supplemental 6. Landmark Analyses

Landmark analyses were performed at 3, 6, 12, and 18 months post-transplant. At each landmark, at-risk patients (i.e., alive without allograft loss) were classified by their dd-cfDNA elevation status at that time point, and the association with subsequent allograft loss was evaluated from the landmark forward.

Supplemental Table 5. Landmark Analysis to Estimate the HR of 3-Year Allograft Loss at 3-, 6-, 12-, and 18-Month Post-Transplant*

dd-cfDNA Status	Cause-specific HR	95% CI
**3-Month Landmark Analysis**		
Intermediate vs. Low	1.70	[0.24, 12.23]
High vs. Low	3.16	[1.47, 6.80]
**6-Month Landmark Analysis**		
Intermediate vs. Low	1.30	[0.31, 5.48]
High vs. Low	2.62	[1.15, 5.96]
**12-Month Landmark Analysis**		
Intermediate vs. Low	1.77	[0.51, 6.19]
High vs. Low	3.46	[1.52, 7.88]
**18-Month Landmark Analysis**		
Intermediate vs. Low	1.45	[0.41, 5.10]
High vs. Low	2.46	[1.02, 5.92]

** Death without graft loss was modeled as a competing risk. Low dd-cfDNA group is the reference category at each landmark.*

Supplemental 7. Causes of Allograft Loss

The causes of allograft loss were evaluated across dd-cfDNA trajectory groups (Low, Intermediate, and High). A total of 35 patients (2.8%) experienced graft loss during follow-up. Rejection was the predominant cause, accounting for 65.7% of all cases, followed by infection (14.3%) and recurrent disease (11.4%). The distribution of etiologies differed across dd-cfDNA groups, with rejection being most frequent among patients in the Intermediate and High groups.

Supplemental Table 6. Causes of Allograft Loss by dd-cfDNA Group

Cause of Allograft Loss	Total (N = 35)	Low (N = 10)	Intermediate (N = 8)	High (N = 17)
Surgical	1 (2.9%)	1 (10.0%)	0 (0%)	0 (0%)
Acute Kidney Injury (AKI)	1 (2.9%)	0 (0%)	0 (0%)	1 (5.9%)
Rejection	23 (65.7%)	5 (50.0%)	6 (75.0%)	12 (70.6%)
Recurrence of Disease	4 (11.4%)	2 (20.0%)	1 (12.5%)	1 (5.9%)
Infection	5 (14.3%)	2 (20.0%)	0 (0%)	3 (17.6%)
Multiple Organ Failure	1 (2.9%)	0 (0%)	1 (12.5%)	0 (0%)
